# Longitudinal Assessment of Antimicrobial Susceptibility among Gram-Negative and Gram-Positive Organisms Collected from Italy as Part of the Tigecycline Evaluation and Surveillance Trial between 2004 and 2011

**DOI:** 10.3390/ph6111381

**Published:** 2013-11-07

**Authors:** Stefania Stefani, Michael J. Dowzicky

**Affiliations:** 1 Department of Bio-Medical Sciences, University of Catania, Via Androne 81, Catania 95124, Italy; 2 Pfizer Inc, Collegeville, PA 19426, USA

**Keywords:** tigecycline, Italy, surveillance, antimicrobial resistance

## Abstract

The Tigecycline Evaluation and Surveillance Trial (T.E.S.T.) was initiated in 2004 to longitudinally monitor the activity of the broad-spectrum glycylcycline antimicrobial tigecycline, and a suite of comparator agents, against an array of clinically important bacterial pathogens worldwide. In this report, we examine the activity of tigecycline and comparators against a collection of 13,245 clinical isolates, both Gram-positive (n = 4,078 and Gram-negative (n = 9,167), collected from 27 centres in Italy between 2004 and 2011. Susceptibility was established according to Clinical Laboratory Standards Institute guidelines. Tigecycline and linezolid exhibited very good activity against Gram-positive pathogens, with MIC_90_s ranging from 0.06 to 0.25 mg/L and 1–4 mg/L, respectively; vancomycin and the carbapenems also showed good activity against select Gram-positive pathogens. Tigecycline was the most active agent against Gram-negative pathogens (except *P. aeruginosa*), with MIC_90_s ranging from 0.25–2 mg/L (16 mg/L for *P. aeruginosa*). Amikacin and the carbapenems also possessed good activity against many Gram-negative pathogens here. ESBL-positive *E. coli* increased in prevalence from 2004 to 2011, while ESBL-positive *Klebsiella* spp., vancomycin-resistant enterococci and MRSA decreased in prevalence. Linezolid, tigecycline and vancomycin susceptibility were very stable over the course of this study, while susceptibility to ampicillin, piperacillin-tazobactam, ceftriaxone and levofloxacin varied over time according to pathogen; minocycline and cefepime susceptibility among several pathogens decreased during this study.

## 1. Introduction

The resistance of Gram-negative and Gram-positive organisms to antimicrobial agents has been widely documented in Europe. The recent report of the European Centre for Disease Prevention and Control (ECDC) included data from 35 Italian hospitals, most of whom used the Clinical and Standards Institute (CLSI) guidelines [1], and it showed that Italy had experienced decreased vancomycin resistance in *Enterococcus faecium* and reduced penicillin non-susceptibility in *Streptococcus pneumoniae* between 2007 and 2011. There were also stable proportions of methicillin-resistant *Staphylococcus aureus* (MRSA) (33–40%), high-level aminoglycoside-resistant *Enterococcus faecalis* (36–50%) and a sudden increase of carbapenem-resistant *Klebsiella pneumoniae* (from 1% in 2006 to 27% in 2011) in Italy during this time.

Among the different surveillance studies run globally, the Tigecycline Evaluation and Surveillance Trial (T.E.S.T.), which has been ongoing since 2004, was initiated to monitor continuously the antibacterial activity of tigecycline and to compare its potency with those of other antimicrobials used in therapy. Tigecycline is the first member of the glycylcycline family of antimicrobials to be administered for bacterial infections. This agent has demonstrated activity against key resistant Gram-negative and Gram-positive phenotypes, both *in vitro* and *in vivo* [2]. This paper will describe the antimicrobial susceptibilities of several important Gram-negative and Gram-positive pathogens, collected in Italy as a part of T.E.S.T., to a range of antimicrobial agents between 2004 and 2011.

## 2. Materials and Methods

### 2.1. Isolate Collection

A total of 27 centres in Italy collected isolates for T.E.S.T. between 2004 and 2011 (2004, six centres; 2005, eight; 2006, 15; 2007, 13; 2008, 17; 2009, 13; 2010, 13; and 2011, 12 centres). A minimum of 135 Gram-negative isolates were required to be submitted by each centre, including 15 *Acinetobacter* spp., 25 *Enterobacter* spp., 25 *Escherichia coli*, 15 *Haemophilus influenzae*, 25 *Klebsiella* spp. (*K. oxytoca* and *K. pneumoniae*), 20 *Pseudomonas aeruginosa*, and 10 *Serratia* spp. All isolates were drawn from samples collected for diagnosis purposes. Isolates of *Enterobacter* spp., *E. coli* and *Klebsiella* spp. were tested for extended-spectrum β-lactamase (ESBL) production. Isolates of *H. influenzae* were tested for β-lactamase (BL) production. Each centre in Italy was also obliged to collect a minimum of 65 Gram-positive isolates, consisting of 15 *Enterococcus* spp. (*E. faecium* and *E. faecalis*), 25 *S. aureus*, 10 *Streptococcus agalactiae*, and 15 *S. pneumoniae*. All body sites were accepted sources of clinical isolates, however, no more than 25% of isolates could be urinary. Only one isolate from each patient could be included in the study, and the participant’s medical history, antimicrobial use, age and gender were not considered.

### 2.2. Antimicrobial Susceptibility Testing

Each participating T.E.S.T. centre was responsible for the initial identification and susceptibility testing of all isolates collected. The minimum inhibitory concentrations (MICs) for each T.E.S.T. agent and pathogen were determined using CLSI broth microdilution methodology [3] and either MicroScan^®^ panels (Dade Microscan Inc., West Sacramento, CA, USA) or Sensititre^®^ plates (TREK Diagnostic Systems, East Grinstead, UK), both of which are compatible with CLSI methodology [4]. The antimicrobial agents that formed the core T.E.S.T. panel for all organisms were: amoxicillin-clavulanate (AMC), ampicillin (AMP), ceftriaxone (CRO), imipenem (IPM), levofloxacin (LVX), meropenem (MEM), minocycline (MIN), piperacillin-tazobactam (TZP) and tigecycline (TGC). Due to reliability and quality control issues with MicroScan^®^ plates containing imipenem, their use was discontinued in 2006 in favour of Sensititre^®^ plates containing meropenem.

Gram-negative pathogens were tested against the core T.E.S.T. agents, plus amikacin (AMK) and cefepime (FEP). Isolates of *A. baumannii* and *P. aeruginosa* were also tested against ceftazidime (CAZ). Gram-positive isolates were tested against linezolid (LZD), penicillin (PEN) and vancomycin (VAN) in addition to the core panel of agents. As well as these, *S. pneumoniae* isolates were tested against the macrolides (azithromycin [AZM], clarithromycin [CLR] and erythromycin [ERY]) plus clindamycin (CLI). The following quality control Gram-negative and Gram-positive strains were tested on each day of isolate testing: *E. coli* ATCC 25922, *H. influenzae* ATCC 49247 and ATCC 49766, *P. aeruginosa* ATCC 27853, *E. faecalis* ATCC 29212, *S. aureus* ATCC 29213, and *S. pneumoniae* ATCC 49619. MIC_90_ data were included in this manuscript only if the daily quality control (QC) test results were within the acceptable range published by the CLSI [5].

After identification and determination of antimicrobial MICs at each T.E.S.T. centre, isolates were sent to a central laboratory, Laboratories International for Microbiology Studies, which is a division of International Health Management Associates, Inc. (IHMA, Schaumburg, IL, USA). The central laboratory organised the transport of isolates from all centres, as well as the storage of isolates. IHMA also performed QC checks on approximately 10% of isolates, which included verification of identification as well as susceptibility testing.

Antimicrobial susceptibility was reported using CLSI breakpoints [5]. For *Enterobacter* spp., the carbapenem breakpoints were revised in 2010 [6]. For tigecycline, the breakpoints as approved by the US Food and Drug Administration in the tigecycline package insert were used in this analysis [7]. As *Acinetobacter* and *Pseudomonas* are not among the organisms listed under the approved clinical indications in the tigecycline prescribing information, no tigecycline breakpoints are available for these organisms [7]. Multidrug resistance (MDR) has previously been defined in numerous different ways [8,9,10], particularly for *P. aeruginosa* and *A. baumannii*; in the current study, as in previous T.E.S.T. reports, MDR is defined as resistance to three or more classes of agents on the T.E.S.T. panel. The class definitions and corresponding agents used in this analysis were aminoglycosides (amikacin), β-lactams (cefepime, ceftazidime, ceftriaxone, or piperacillin-tazobactam), carbapenems (imipenem or meropenem), fluoroquinolones (levofloxacin), and tetracyclines (minocycline). Intermediate resistant isolates were not included in the resistant category in this report.

## 3. Results

A total of 13,245 isolates were submitted from Italian medical centres for T.E.S.T. between 2004 and 2011. Of these, 9,167 (69.2%) isolates were Gram-negative (Table 1 and Table 2) and 4,078 (30.8%) isolates were Gram-positive (Table 1 and Table 3). Imipenem results are available only between 2004 and 2006, thus cannot be discussed in longitudinal terms in this study.

### 3.1. Gram-Negative Isolates

The majority of Gram-negative isolates were *E. coli* (21.1%), followed by *Enterobacter* spp. (18.9%), *P. aeruginosa* (16.0%), *K. pneumoniae* (15.2%), *A. baumannii* (9.6%), *H. influenzae* (8.1%), *S. marcescens* (7.1%), and *K. oxytoca* (4.1%; Table 1). In broad terms, tigecycline, the carbapenems and amikacin were active against most Gram-negative pathogens in Italy during the study period.

#### 3.1.1. *A.* *baumannii*

No breakpoints were available for tigecycline, although the lowest overall MIC_90_ was observed for tigecycline (2 mg/L; Table 2). *A. baumannii* were most susceptible to minocycline with 81.0% susceptibility over all study years combined. Isolates of *A. baumannii* from Italy had low susceptibility (<40%) to most of the remaining T.E.S.T. panel agents: amikacin, cefepime, ceftazidime, ceftriaxone, levofloxacin, meropenem and piperacillin-tazobactam. The susceptibility of isolates to amikacin, cefepime, ceftazidime, ceftriaxone and levofloxacin all increased in 2005 before declining again from 2006 to 2011. From 2006 onwards, there was an overall increase in the resistance of *A. baumannii* isolates to amikacin, cefepime, ceftazidime, ceftriaxone, levofloxacin, and piperacillin-tazobactam (Table 2). Very few *A. baumannii* isolates (<1%) were resistant to minocycline between 2004 and 2007, but this percentage rose to 7.9% and 7.6%, respectively, in 2009 and 2010 before decreasing to 2.5% in 2011. A total of 526 MDR *A. baumannii* isolates were collected in Italy during T.E.S.T. (Table 4), which represented 60.0% of all *A. baumannii* isolates. Over the surveillance period, MDR *A. baumannii* isolates had ≥79.5% resistance to all agents tested with the exception of minocycline (5.1%). Among *A. baumannii*, 79.7% were susceptible to imipenem (Table 2) while 49.0% of MDR isolates were susceptible to imipenem (Table 4).

#### 3.1.2. *Enterobacter* spp.

Isolates of *Enterobacter* spp. were >90% susceptible to amikacin, cefepime, meropenem and tigecycline during T.E.S.T. (Table 2). Susceptibility to minocycline almost halved between 2005 and 2009 (from 85.8% to 49.8%), then doubled again to 82.8% in 2011. Almost 50% of isolates were resistant to ceftriaxone over all T.E.S.T. years combined, with the highest resistance recorded in 2004 (64.8%) and the lowest in 2011 (26.3%). The lowest resistance over all T.E.S.T. years was to tigecycline (0.9%). Among *Enterobacter* isolates, 86.9% were susceptible to imipenem between 2004 and 2006 (Table 2).

**Table 1:** Numbers of Gram-negative and Gram-positive isolates collected in Italy during Tigecycline Evaluation and Surveillance Trial (T.E.S.T.) 2004–2011.

Organism	2004	2005	2006	2007	2008	2009	2010	2011	2004–2011
	N	N	N	N	N	N	N	N	N
Gram-negative									
*A. baumannii*	47 (46/1)	51 (46/5)	116 (61/55)	77	193	127	145	121	877 (153/724)
*Enterobacter* spp	91 (89/2)	120 (113/7)	252 (156/96)	176	391	213	276	209	1728 (358/1370)
*E. coli*	91 (89/2)	139 (138/1)	254 (148/106)	190	432	252	285	288	1931 (375/1556)
*H. influenzae*	45 (44/1)	71 (48/23)	109 (71/38)	75	155	115	80	89	739 (163/576)
*K. oxytoca*	16 (14/2)	29	67 (42/25)	46	73	69	41	39	380 (85/295)
*K. pneumoniae*	77 (74/3)	86 (80/6)	191 (117/74)	127	329	200	225	160	1395 (271/1124)
*P. aeruginosa*	72 (70/2)	103 (97/6)	186 (111/75)	144 (1/143)	352	188	222	202	1469 (279/1190)
*S. marcescens*	42 (38/4)	42 (38/4)	90 (50/40)	67	140	75	95	97	648 (126/522)
**Total**	481 (464/17)	641 (589/52)	1265 (756/509)	902 (1/901)	2065	1239	1369	1205	9167 (1810/7357)
**Gram-positive**									
*E. faecalis*	32 (29/3)	52 (42/10)	106 (55/51)	84	132	92	115	127	740 (126/614)
*E. faecium*	22 (20/2)	16 (11/5)	40 (26/14)	19	103	50	45	41	336 (57/279)
*S. aureus*	91 (83/8)	113 (100/13)	217 (89/128)	187	376	224	278	234	1720 (272/1448)
*S. agalactiae*	36	40 (21/19)	91 (38/53)	72	133	76	106	58	612 (95/517)
*S. pneumoniae*	41 (15/26)	65 (48/17)	111 (59/52)	84	143	105	92	29	670 (122/548)
**Total**	222 (183/39)	286 (222/64)	565 (267/298)	446	887	547	636	489	4078 (672/3406)
**All Isolates Total**	703 (647/56)	927 (811/116)	1830 (1023/807)	1348 (1/1347)	2952	1786	2005	1694	13245 (2482/10763)

N = total number of isolates; values given in parentheses refer to the number of isolates tested against imipenem and meropenem, respectively; where no parentheses are given, all isolates were tested against imipenem (2004–2006) or meropenem (2007–2011).

**Table 2:** Minimum inhibitory concentration (MIC)_90_ (mg/L), antimicrobial susceptibility (%S), and antimicrobial resistance (%R) for Gram-negative isolates collected in Italy during T.E.S.T. 2004–2011.

Organism	Agent^a^	2004			2005			2006			2007			2008			2009			2010			2011			2004–2011		
		MIC_90_	%S	%R	MIC_90_	%S	%R	MIC_90_	%S	%R	MIC_90_	%S	%R	MIC_90_	%S	%R	MIC_90_	%S	%R	MIC_90_	%S	%R	MIC_90_	%S	%R	MIC_90_	%S	%R
*A. baumannii*	TGC	1	NA	NA	1	NA	NA	2	NA	NA	1	NA	NA	2	NA	NA	2	NA	NA	2	NA	NA	1	NA	NA	2	NA	NA
	AMP	≥64	NA	NA	≥64	NA	NA	≥64	NA	NA	≥64	NA	NA	≥64	NA	NA	≥64	NA	NA	≥64	NA	NA	≥64	NA	NA	≥64	NA	NA
	AMC	≥64	NA	NA	≥64	NA	NA	≥64	NA	NA	≥64	NA	NA	≥64	NA	NA	≥64	NA	NA	≥64	NA	NA	≥64	NA	NA	≥64	NA	NA
	TZP	≥256	63.8	31.9	≥256	60.8	29.4	≥256	29.3	55.2	≥256	39.0	57.1	≥256	29.5	63.7	≥256	27.6	67.7	≥256	20.0	75.9	≥256	17.4	81.0	≥256	30.4	63.3
	CAZ	≥64	42.6	53.2	≥64	52.9	41.2	≥64	23.3	71.6	≥64	39.0	59.7	≥64	30.6	65.3	≥64	26.0	70.9	≥64	18.6	75.2	32	20.7	75.2	≥64	28.3	67.4
	CRO	≥128	25.5	53.2	≥128	39.2	35.3	≥128	16.4	75.9	≥128	33.8	59.7	≥128	18.7	70.5	≥128	18.1	73.2	≥128	13.8	77.9	64	15.7	74.4	≥128	20.0	69.4
	FEP	≥64	40.4	36.2	32	49.0	27.5	≥64	24.1	57.8	≥64	40.3	53.2	≥64	32.6	57.5	≥64	35.4	51.2	≥64	26.2	60.0	≥64	18.2	63.6	≥64	30.9	54.6
	IPM	≥32	82.6	17.4	8	82.6	8.7	16	75.4	14.8	-	-	-	-	-	-	-	-	-	-	-	-	-	-	-	16	79.7	13.7
	MEM	-	-	-	-	-	-	≥32	30.9	47.3	16	63.6	28.6	≥32	48.7	37.3	≥32	36.2	53.5	≥32	25.5	69.0	≥32	19.0	79.3	≥32	36.9	53.6
	LVX	≥16	53.2	40.4	≥16	60.8	27.5	≥16	25.0	63.8	≥16	36.4	55.8	≥16	30.6	66.3	≥16	26.8	69.3	≥16	20.0	76.6	≥16	15.7	84.3	≥16	29.0	66.0
	AMK	≥128	55.3	31.9	64	76.5	21.6	≥128	43.1	49.1	≥128	42.9	54.5	≥128	43.0	51.8	≥128	30.7	65.4	≥128	37.2	60.0	≥128	20.7	73.6	≥128	39.8	55.2
	MIN	2	100	0.0	1	96.1	0.0	2	96.6	0.9	1	100	0.0	8	88.6	2.1	8	71.7	7.9	8	55.2	7.6	8	68.6	2.5	8	81.0	3.3
*Enterobacter* spp.	TGC	4	87.9	2.2	2	97.5	0.8	4	86.5	2.0	1	97.7	0.0	2	96.7	0.3	2	94.4	1.9	2	95.7	1.1	1	99.0	0.0	2	94.7	0.9
	AMP	≥64	0.0	95.6	≥64	0.8	94.2	≥64	0.0	94.0	≥64	1.2	91.8	≥64	2.8	88.2	≥64	5.2	83.6	≥64	1.5	85.1	≥64	1.4	86.1	≥64	1.9	88.9
	AMC	≥64	6.6	90.1	≥64	4.2	94.2	≥64	2.4	95.2	≥64	5.1	86.4	≥64	3.6	92.3	≥64	5.6	92.0	≥64	2.5	95.3	≥64	2.4	96.2	≥64	3.7	93.1
	TZP	≥256	58.2	31.9	128	63.3	17.5	≥256	62.3	23.0	≥256	65.9	23.3	≥256	63.9	25.1	≥256	62.4	23.0	≥256	60.5	23.9	64	82.3	9.6	≥256	65.0	22.1
	CRO	≥128	29.7	64.8	≥128	45.8	52.5	≥128	49.2	48.0	≥128	55.7	39.8	≥128	49.6	48.6	≥128	52.1	45.5	≥128	50.0	47.5	64	70.3	26.3	≥128	51.7	45.5
	FEP	≥64	75.8	16.5	8	93.3	4.2	16	86.9	8.7	8	90.3	8.0	8	91.6	6.1	8	92.5	5.2	16	88.4	9.1	4	94.7	2.9	8	90.0	7.1
	IPM	2	79.8	3.4	4	80.5	10.6	1	95.5	3.9	-	-	-	-	-	-	-	-	-	-	-	-	-	-	-	2	86.9	5.9
	MEM	-	-	-	-	-	-	1	93.8	6.3	0.5	96.0	2.3	0.5	95.9	2.8	0.25	98.1	1.9	0.5	96.7	1.8	0.12	99.0	0.5	0.5	96.6	2.4
	LVX	≥16	64.8	31.9	≥16	75.0	24.2	≥16	75.4	22.2	≥16	77.3	22.2	≥16	77.0	22.0	≥16	82.6	16.0	≥16	76.1	23.2	2	90.9	7.7	≥16	78.2	20.4
	AMK	16	95.6	0.0	8	95.8	2.5	8	96.4	3.2	4	97.2	1.1	8	95.9	1.5	2	97.7	2.4	4	98.6	0.7	4	99.0	0.0	8	97.1	1.5
	MIN	16	68.1	12.1	8	85.8	5.8	16	78.2	11.9	16	75.6	10.8	16	68.5	12.5	16	49.8	13.6	16	51.8	19.2	8	82.8	5.7	16	68.6	12.2
*E. coli*	TGC	0.5	100	0.0	0.5	100	0.0	0.5	100	0.0	0.5	100	0.0	0.5	100	0.0	0.5	99.6	0.0	1	100	0.0	0.5	100	0.0	0.5	99.9	0.0
	AMP	≥64	29.7	70.3	≥64	46.8	53.2	≥64	30.3	68.5	≥64	35.8	63.7	≥64	23.6	75.7	≥64	27.0	71.0	≥64	22.8	76.8	≥64	26.7	72.2	≥64	28.4	70.7
	AMC	32	53.8	27.5	32	72.7	12.2	32	62.2	13.4	16	65.8	8.4	32	54.4	18.8	32	52.8	22.6	32	48.1	21.4	32	71.9	10.4	32	59.3	16.6
	TZP	128	83.5	13.2	64	87.1	8.6	32	89.0	5.1	16	92.1	2.6	64	82.2	8.6	128	77.8	11.5	128	78.2	10.5	32	88.9	6.6	64	84.3	8.1
	CRO	64	79.1	18.7	≥128	81.3	17.3	≥128	76.0	22.8	≥128	83.2	16.8	≥128	63.4	35.6	≥128	58.7	39.3	≥128	57.5	41.8	64	64.6	34.4	≥128	67.7	31.2
	FEP	8	93.4	4.4	32	87.1	11.5	16	88.6	7.9	32	85.3	12.6	≥64	77.8	17.4	≥64	74.2	23.8	≥64	72.3	20.4	≥64	77.4	16.3	≥64	80.0	15.7
	IPM	0.5	100	0.0	0.5	100	0.0	0.25	98.6	0.0	-	-	-	-	-	-	-	-	-	-	-	-	-	-	-	0.5	99.5	0.0
	MEM	-	-	-	-	-	-	≤0.06	99.1	0.9	≤0.06	100	0.0	≤0.06	97.9	1.2	≤0.06	98.4	0.4	≤0.06	99.6	0.4	≤0.06	100	0.0	≤0.06	99.0	0.5
	LVX	≥16	63.7	33.0	≥16	67.6	30.2	≥16	57.5	39.8	≥16	63.7	33.7	≥16	46.1	51.4	≥16	52.0	47.2	≥16	42.1	56.1	≥16	50.3	43.8	≥16	52.5	44.7
	AMK	8	100	0.0	16	94.2	3.6	8	98.4	0.8	8	98.9	0.5	8	96.3	2.3	8	96.8	1.2	8	98.2	0.4	8	99.0	0.0	8	97.6	1.1
	MIN	≥32	70.3	23.1	16	77.7	10.8	16	69.3	19.7	16	73.7	13.2	16	74.5	16.0	≥32	70.2	19.0	16	62.8	20.0	16	81.9	10.8	16	72.6	16.4
*H. influenzae*	TGC	0.25	100	NA	0.25	98.6	NA	0.25	94.5	NA	0.25	92.0	NA	0.25	99.4	NA	0.25	100	NA	0.25	97.5	NA	0.25	100	NA	0.25	97.8	NA
	AMP	2	88.9	8.9	16	81.7	14.1	2	87.2	9.2	16	84.0	14.7	8	84.5	14.2	≤0.5	96.5	2.6	32	85.0	15.0	1	91.0	6.7	4	87.6	10.6
	AMC	1	100	0.0	2	95.8	4.2	1	100	0.0	1	100	0.0	1	100	0.0	1	100	0.0	2	100	0.0	1	100	0.0	1	99.6	0.4
	TZP	≤0.06	100	0.0	0.12	98.6	1.4	≤0.06	99.1	0.9	≤0.06	100	0.0	≤0.06	100	0.0	≤0.06	100	0.0	≤0.06	100	0.0	≤0.06	100	0.0	≤0.06	99.7	0.3
	CRO	≤0.06	100	NA	0.12	98.6	NA	≤0.06	100	NA	0.12	100	NA	≤0.06	100	NA	≤0.06	100	NA	≤0.06	100	NA	≤0.06	100	NA	≤0.06	99.9	NA
	FEP	≤0.5	100	NA	≤0.5	98.6	NA	≤0.5	100	NA	≤0.5	94.7	NA	≤0.5	99.4	NA	≤0.5	100	NA	≤0.5	100	NA	≤0.5	98.9	NA	≤0.5	99.1	NA
	IPM	1	100	NA	1	100	NA	0.5	100	NA	-	-	-	-	-	-	-	-	-	-	-	-	-	-	-	1	100	NA
	MEM	-	-	-	0.25	100	NA	0.12	100	NA	0.12	100	NA	0.25	100	NA	0.12	100	NA	0.12	100	NA	0.25	100	NA	0.12	100	NA
	LVX	0.015	100	NA	0.12	100	NA	0.03	100	NA	0.03	100	NA	0.03	100	NA	0.015	100	NA	0.03	100	NA	0.03	100	NA	0.03	100	NA
	AMK	8	NA	NA	8	NA	NA	8	NA	NA	8	NA	NA	8	NA	NA	8	NA	NA	8	NA	NA	8	NA	NA	8	NA	NA
	MIN	1	97.8	2.2	2	94.4	0.0	1	99.1	0.0	1	98.7	0.0	2	98.7	0.0	1	99.1	0.9	1	100	0.0	1	95.5	2.3	1	98.1	0.5
*K. oxytoca*	TGC	-	-	-	2	96.6	0.0	1	97.0	0.0	1	97.8	0.0	0.5	98.6	0.0	1	97.1	0.0	1	95.1	0.0	0.5	100	0.0	1	97.4	0.0
	AMP	-	-	-	≥64	0.0	100	≥64	0.0	92.5	≥64	2.2	89.1	≥64	1.4	87.7	≥64	2.9	82.6	≥64	2.4	87.8	≥64	15.4	64.1	≥64	2.9	86.3
	AMC	-	-	-	16	75.9	6.9	32	77.6	13.4	32	80.4	13.0	32	71.2	12.3	32	73.9	17.4	16	85.4	9.8	4	92.3	5.1	32	77.1	13.2
	TZP	-	-	-	≥256	82.8	13.8	≥256	85.1	14.9	4	95.7	4.4	≥256	79.5	17.8	≥256	82.6	15.9	8	90.2	9.8	8	94.9	5.1	≥256	85.0	13.9
	CRO	-	-	-	8	82.8	13.8	8	85.1	11.9	0.5	93.5	6.5	32	76.7	21.9	16	73.9	24.6	16	87.8	12.2	0.25	100	0.0	8	82.6	16.1
	FEP	-	-	-	2	93.1	6.9	4	97.0	1.5	≤0.5	100	0.0	8	95.9	2.7	4	95.7	1.5	≤0.5	97.6	2.4	≤0.5	100	0.0	2	97.1	1.8
	IPM	-	-	-	0.5	100	0.0	0.5	100	0.0	-	-	-	-	-	-	-	-	-	-	-	-	-	-	-	0.5	98.8	0.0
	MEM	-	-	-	-	-	-	0.12	100	0.0	≤0.06	100	0.0	≤0.06	100	0.0	≤0.06	98.6	0.0	0.12	100	0.0	≤0.06	100	0.0	0.12	99.7	0.0
	LVX	-	-	-	8	86.2	13.8	0.25	95.5	4.5	0.5	95.7	2.2	4	89.0	9.6	4	89.9	8.7	0.5	92.7	7.3	0.12	100	0.0	0.5	92.6	6.6
	AMK	-	-	-	8	100	0.0	4	98.5	1.5	4	100	0.0	4	97.3	1.4	4	100	0.0	2	100	0.0	4	100	0.0	4	99.2	0.5
	MIN	-	-	-	16	86.2	10.3	8	88.1	7.5	16	84.8	10.9	8	84.9	6.9	8	76.8	2.9	8	78.0	9.8	8	84.6	5.1	8	83.7	6.8
*K. pneumoniae*	TGC	2	92.2	0.0	1	100	0.0	2	92.7	1.1	1	99.2	0.0	1	94.8	1.5	2	96.5	2.0	2	95.6	0.4	2	97.5	0.0	2	95.8	0.9
	AMP	≥64	0.0	94.8	≥64	1.2	82.6	≥64	0.0	89.4	≥64	0.8	93.7	≥64	0.9	90.0	≥64	1.0	91.0	≥64	2.2	90.7	≥64	5.6	89.4	≥64	1.5	90.2
	AMC	32	59.7	22.1	32	70.9	17.4	32	71.7	17.8	32	69.3	18.1	32	59.9	22.5	≥64	53.0	35.5	≥64	48.0	37.3	≥64	56.3	28.1	≥64	59.7	26.0
	TZP	128	77.9	15.6	64	82.6	5.8	≥256	80.6	16.8	≥256	78.0	17.3	≥256	70.5	21.6	≥256	63.0	25.5	≥256	57.8	33.8	≥256	68.8	25.6	≥256	70.4	22.2
	CRO	≥128	58.4	41.6	64	73.3	25.6	64	78.0	20.4	≥128	66.1	32.3	≥128	63.5	34.0	≥128	60.0	37.5	≥128	51.6	48.0	64	56.3	43.1	≥128	62.8	35.7
	FEP	16	84.4	9.1	16	89.5	7.0	16	86.9	9.4	≥64	78.0	19.7	≥64	77.2	20.4	≥64	73.5	23.5	≥64	59.6	38.7	≥64	68.8	27.5	≥64	75.4	21.6
	IPM	1	98.6	0.0	0.5	98.8	0.0	0.5	91.5	0.9	-	-	-	-	-	-	-	-	-	-	-	-	-	-	-	0.5	95.6	0.4
	MEM	-	-	-	-	-	-	0.12	98.6	1.4	2	87.4	2.4	0.5	92.7	2.7	2	86.5	9.0	≥32	80.4	16.4	≥32	85.0	14.4	2	87.9	8.1
	LVX	8	77.9	11.7	8	84.9	14.0	≥16	85.9	12.6	≥16	76.4	22.0	≥16	76.3	21.6	≥16	66.5	33.0	≥16	56.4	42.2	≥16	60.0	35.6	≥16	71.8	25.9
	AMK	16	92.2	0.0	8	94.2	5.8	4	93.2	6.3	16	91.3	8.7	64	89.1	10.3	32	88.5	8.0	32	85.8	6.7	32	88.8	1.9	32	89.7	6.9
	MIN	16	68.8	23.4	16	83.7	11.6	≥32	75.9	16.2	≥32	78.7	16.5	≥32	65.0	29.2	≥32	57.0	23.5	≥32	53.8	29.3	16	62.5	17.5	≥32	65.9	22.7
*P. aeruginosa*	TGC	≥32	NA	NA	≥32	NA	NA	≥32	NA	NA	16	NA	NA	16	NA	NA	≥32	NA	NA	16	NA	NA	16	NA	NA	16	NA	NA
	AMP	≥64	NA	NA	≥64	NA	NA	≥64	NA	NA	≥64	NA	NA	≥64	NA	NA	≥64	NA	NA	≥64	NA	NA	≥64	NA	NA	≥64	NA	NA
	AMC	≥64	NA	NA	≥64	NA	NA	≥64	NA	NA	≥64	NA	NA	≥64	NA	NA	≥64	NA	NA	≥64	NA	NA	≥64	NA	NA	≥64	NA	NA
	TZP	≥256	54.2	30.6	128	69.9	12.6	128	67.2	16.7	≥256	66.7	19.4	≥256	62.2	24.1	≥256	56.9	23.4	≥256	55.0	32.9	128	67.8	17.8	≥256	62.4	22.6
	CAZ	≥64	59.7	27.8	32	69.9	20.4	≥64	62.9	24.7	≥64	73.6	15.3	≥64	61.1	28.1	≥64	55.3	33.0	≥64	55.4	32.9	32	69.8	21.8	≥64	62.7	26.3
	CRO	≥128	NA	NA	≥128	NA	NA	≥128	NA	NA	≥128	NA	NA	≥128	NA	NA	≥128	NA	NA	≥128	NA	NA	64	NA	NA	≥128	NA	NA
	FEP	≥64	44.4	29.2	32	65.0	16.5	≥64	62.4	21.0	32	73.6	13.2	32	66.2	18.8	≥64	66.0	21.8	32	60.8	19.8	32	63.9	20.8	32	64.1	19.7
	IPM	16	58.6	32.9	8	68.0	17.5	16	62.2	27.0	-	-	-	-	-	-	-	-	-	-	-	-	-	-	-	16	63.4	25.1
	MEM	-	-	-	-	-	-	≥32	68.0	25.3	16	64.3	27.3	≥32	65.6	27.0	≥32	63.8	30.9	≥32	57.2	32.9	≥32	59.4	31.7	≥32	62.6	29.6
	LVX	≥16	54.2	43.1	≥16	51.5	40.8	≥16	52.2	40.3	≥16	52.8	38.9	≥16	54.8	39.5	≥16	55.9	37.2	≥16	51.8	40.5	≥16	61.4	35.1	≥16	54.6	39.1
	AMK	16	90.3	5.6	32	88.3	4.9	16	90.9	5.9	16	92.4	2.8	32	87.5	5.7	64	83.0	10.1	32	89.2	7.2	32	86.6	8.9	32	88.2	6.6
	MIN	≥32	NA	NA	≥32	NA	NA	≥32	NA	NA	≥32	NA	NA	≥32	NA	NA	≥32	NA	NA	≥32	NA	NA	≥32	NA	NA	≥32	NA	NA
*S. marcescens*	TGC	2	92.9	0.0	2	100	0.0	2	94.4	0.0	2	100	0.0	2	97.1	0.0	2	97.3	0.0	2	94.7	2.1	2	94.8	2.1	2	96.3	0.6
	AMP	≥64	2.4	95.2	≥64	7.1	81.0	≥64	0.0	96.6	≥64	0.0	82.8	≥64	5.0	83.6	≥64	8.0	69.3	≥64	5.3	72.6	≥64	1.0	75.3	≥64	3.6	81.3
	AMC	≥64	2.4	92.9	≥64	4.8	90.5	≥64	5.6	94.4	≥64	4.5	89.6	≥64	3.6	92.1	≥64	5.3	86.7	≥64	5.3	92.6	≥64	2.1	91.8	≥64	4.2	91.5
	TZP	16	90.5	7.1	8	95.2	0.0	64	77.8	10.0	4	98.5	0.0	16	90.7	2.9	16	92.0	5.3	4	95.8	1.1	8	94.8	3.1	16	91.5	3.7
	CRO	32	64.3	23.8	16	83.3	14.3	32	74.4	23.3	1	94.0	4.5	16	77.9	16.4	32	80.0	20.0	8	81.1	13.7	2	82.5	7.2	16	79.9	15.1
	FEP	8	92.9	7.1	4	100	0.0	2	94.4	4.4	≤0.5	95.5	4.5	1	96.4	2.1	2	94.7	4.0	1	100	0.0	1	99.0	1.0	2	96.8	2.6
	IPM	2	71.1	0.0	1	94.7	2.6	1	90.0	8.0	-	-	-	-	-	-	-	-	-	-	-	-	-	-	-	2	85.7	4.0
	MEM	-	-	-	-	-	-	0.12	100	0.0	0.12	100	0.0	0.5	99.3	0.7	0.12	98.7	1.3	0.25	93.7	4.2	0.25	99.0	1.0	0.25	98.3	1.3
	LVX	1	92.9	4.8	1	97.6	0.0	2	90.0	1.1	2	91.0	1.5	2	91.4	5.7	4	89.3	2.7	2	91.6	6.3	2	96.9	3.1	2	92.3	3.6
	AMK	8	100	0.0	8	97.6	2.4	8	98.9	0.0	4	97.0	0.0	4	96.4	0.7	16	96.0	2.7	4	98.9	1.1	8	95.9	1.0	8	97.4	0.9
	MIN	8	64.3	4.8	4	97.6	2.4	8	84.4	1.1	8	86.6	3.0	16	66.4	11.4	16	54.7	13.3	16	57.9	13.7	16	66.0	12.4	8	70.2	8.8

^a^ AMK = amikacin; AMC = amoxicillin-clavulanate; AMP = ampicillin; FEP = cefepime; CAZ = ceftazidime; CRO = ceftriaxone; IPM = imipenem; LVX = levofloxacin; MEM = meropenem; MIN = minocycline; TZP = piperacillin-tazobactam; TGC = tigecycline. NA = susceptibility or resistance breakpoint not available. - MIC90, %S and %R are not given where N ≤20. No imipenem data was collected after 2006. Susceptible (S), resistance (R) breakpoints (mg/L): *A. baumannii*: TZP, S ≤ 16, R ≥ 128; CAZ, S ≤ 8, R ≥ 32; CRO, S ≤ 8, R ≥ 64; FEP, S ≤ 8, R ≥ 32; IPM, S≤= 4, R ≥ 16; MEM, S ≤ 4, R ≥ 16; LVX, S≤= 2, R ≥ 8; AMK, S ≤ 16, R ≥ 64; MIN, S ≤ 4, R ≥ 16; *Enterobacter* spp.: TGC, S ≤ 2, R ≥ 8; AMP, S ≤ 8, R ≥ 32; AMC, S ≤ 8, R ≥ 32; TZP, S ≤ 16, R ≥ 128; CRO, S ≤ 1, R ≥ 4; FEP, S ≤ 8, R ≥ 32; IPM, S ≤ 1, R ≥ 4; MEM, S ≤ 1, R ≥ 4; LVX, S ≤ 2, R ≥ 8; AMK, S ≤ 16, R ≥ 64; MIN, S ≤ 4, R ≥ 16; *E. coli*: TGC, S ≤ 2, R ≥ 8; AMP, S ≤ 8, R ≥ 32; AMC, S ≤ 8, R ≥ 32; TZP, S ≤ 16, R ≥ 128; CRO, S ≤ 1, R ≥ 4; FEP, S ≤ 8, R ≥ 32; IPM, S ≤ 1, R ≥ 4; MEM, S ≤ 1, R ≥ 4; LVX, S ≤ 2, R ≥ 8; AMK, S ≤ 16, R ≥ 64; MIN, S ≤ 4, R ≥ 16; *H. influenza*: TIG, S ≤ 0.25; AMP, S ≤ 1, R ≥ 4; AMC, S ≤ 4, R ≥ 8; CRO, S ≤ 2; FEP, S ≤ 2; IPM, S ≤ 4; MEM, S ≤ 0.5; LVX, S ≤ 2; MIN, S ≤ 2, R ≥ 8; *Klebsiella* spp.: TIG, S ≤ 2, R ≥ 8; AMP, S ≤ 8, R ≥ 32; AMC, S ≤ 8, R ≥ 32; TZP, S ≤ 16, R ≥ 128; CRO, S ≤ 1, R ≥ 4; FEP, S ≤ 8, R ≥ 32; IPM, S ≤ 1, R ≥ 4; MEM, S ≤ 1, R ≥ 4; LVX, S ≤ 2, R ≥ 8; AMK, S ≤ 16, R ≥ 64; MIN, S ≤ 4, R ≥ 16; *P. aeruginosa*: TZP, S ≤ 16, R ≥ 128; CAZ, S ≤ 8, R ≥ 32; FEP, S ≤ 8, R ≥ 32; IPM, S ≤ 2, R ≥ 8; MEM, S ≤ 2, R ≥ 8; LVX, S ≤ 2, R ≥ 8; AMK, S ≤ 16, R ≥ 64; *S. marcescens*: TGC, S ≤ 2, R ≥ 8; AMP, S ≤ 8, R ≥ 32; AMC, S ≤ 8, R ≥ 32; TZP, S ≤ 16, R ≥ 128; CRO, S ≤ 1, R ≥ 4; FEP, S ≤ 8, R ≥ 32; IPM, S ≤ 1, R ≥ 4; MEM, S ≤ 1, R ≥ 4; LVX, S ≤ 2, R ≥ 8; AMK, S ≤ 16, R ≥ 64; MIN, S ≤ 4, R ≥ 16.

**Table 3:** MIC_90_ (mg/L), antimicrobial susceptibility (%S), and antimicrobial resistance (%R) for Gram-positive isolates collected in Italy during T.E.S.T. 2004–2011.

Organism	Agent^a^	2004			2005			2006			2007			2008			2009			2010			2011			2004–2011		
		MIC_90_	%S	%R	MIC_90_	%S	%R	MIC_90_	%S	%R	MIC_90_	%S	%R	MIC_90_	%S	%R	MIC_90_	%S	%R	MIC_90_	%S	%R	MIC_90_	%S	%R	MIC_90_	%S	%R
*E. faecalis*	TGC	0.25	96.9	NA	0.25	100	NA	0.25	100	NA	0.25	100	NA	0.25	100	NA	0.25	100	NA	0.25	99.1	NA	0.12	100	NA	0.25	99.7	NA
	AMP	2	100	0.0	1	100	0.0	2	100	0.0	2	100	0.0	2	100	0.0	2	100	0.0	2	97.4	2.6	1	100	0.0	2	99.6	0.4
	PEN	8	100	0.0	4	100	0.0	4	100	0.0	4	100	0.0	8	100.0	0.0	8	100	0.0	8	97.4	2.6	4	100	0.0	8	99.6	0.4
	AMC	2	NA	NA	1	NA	NA	1	NA	NA	1	NA	NA	2	NA	NA	2	NA	NA	1	NA	NA	1	NA	NA	1	NA	NA
	TZP	8	NA	NA	8	NA	NA	8	NA	NA	8	NA	NA	8	NA	NA	16	NA	NA	8	NA	NA	4	NA	NA	8	NA	NA
	CRO	≥128	NA	NA	≥128	NA	NA	≥128	NA	NA	≥128	NA	NA	≥128	NA	NA	≥128	NA	NA	≥128	NA	NA	≥128	NA	NA	≥128	NA	NA
	IPM	4	NA	NA	2	NA	NA	8	NA	NA	-	-	-	-	-	-	-	-	-	-	-	-	-	-	-	8	NA	NA
	MEM	-	-	-	8	NA	NA	16	NA	NA	16	NA	NA	8	NA	NA	16	NA	NA	8	NA	NA	8	NA	NA	8	NA	NA
	LVX	32	56.3	43.8	32	71.2	25.0	≥64	62.3	37.7	32	70.2	29.8	≥64	69.7	29.5	≥64	66.3	28.3	≥64	60.0	39.1	≥64	63.8	34.6	≥64	65.3	33.2
	LZD	2	100	0.0	2	100	0.0	2	100	0.0	2	100	0.0	2	100.0	0.0	2	100	0.0	2	100	0.0	2	100	0.0	2	100	0.0
	MIN	≥16	37.5	25.0	≥16	36.5	13.5	≥16	28.3	28.3	≥16	29.8	42.9	≥16	21.2	47.7	≥16	14.1	58.7	≥16	16.5	61.7	≥16	32.3	12.6	≥16	25.3	38.5
	VAN	≥64	81.3	18.8	2	96.2	3.9	2	97.2	2.8	2	97.6	2.4	2	95.5	3.8	2	96.7	3.3	4	95.7	4.4	2	100	0.0	2	96.4	3.5
*E. faecium*	TGC	0.12	100	NA	-	-	-	0.12	100	NA	-	-	-	0.25	96.1	NA	0.25	100	NA	0.25	100	NA	0.06	100	NA	0.25	98.8	NA
	AMP	≥32	40.9	59.1	-	-	-	≥32	10.0	90.0	-	-	-	≥32	15.5	84.5	≥32	26.0	74.0	≥32	24.4	75.6	≥32	22.0	78.0	≥32	20.2	79.8
	PEN	≥16	36.4	63.6	-	-	-	≥16	7.5	92.5	-	-	-	≥16	31.1	68.9	≥16	22.0	78.0	≥16	35.6	64.4	≥16	53.7	46.3	≥16	29.5	70.5
	AMC	≥16	NA	NA	-	-	-	≥16	NA	NA	-	-	-	≥16	NA	NA	≥16	NA	NA	≥16	NA	NA	≥16	NA	NA	≥16	NA	NA
	TZP	≥32	NA	NA	-	-	-	≥32	NA	NA	-	-	-	≥32	NA	NA	≥32	NA	NA	≥32	NA	NA	≥32	NA	NA	≥32	NA	NA
	CRO	≥128	NA	NA	-	-	-	≥128	NA	NA	-	-	-	≥128	NA	NA	≥128	NA	NA	≥128	NA	NA	≥128	NA	NA	≥128	NA	NA
	IPM	≥32	NA	NA	-	-	-	≥32	NA	NA	-	-	-	-	-	-	-	-	-	-	-	-	-	-	-	≥32	NA	NA
	MEM	-	-	-	-	-	-	≥32	NA	NA	-	-	-	≥32	NA	NA	≥32	NA	NA	≥32	NA	NA	≥32	NA	NA	≥32	NA	NA
	LVX	≥64	27.3	72.7	-	-	-	≥64	15.0	85.0	-	-	-	≥64	6.8	89.3	≥64	14.0	82.0	≥64	13.3	77.8	≥64	12.2	85.4	≥64	12.5	83.0
	LZD	2	100	0.0	-	-	-	2	100	0.0	-	-	-	2	98.1	1.9	2	100	0.0	2	97.8	0.0	2	100	0.0	2	99.1	0.6
	MIN	≥16	68.2	18.2	-	-	-	8	80.0	5.0	-	-	-	≥16	84.5	11.7	≥16	70.0	28.0	≥16	71.1	15.6	4	90.2	2.4	≥16	78.9	13.1
	VAN	≥64	77.3	18.2	-	-	-	≥64	50.0	47.5	-	-	-	≥64	85.4	13.6	≥64	88.0	12.0	≥64	84.4	13.3	1	100	0.0	≥64	82.1	16.4
*S. aureus*	TGC	0.25	100	NA	0.25	100	NA	0.25	100	NA	0.25	100	NA	0.25	100	NA	0.5	100	NA	0.25	100	NA	0.25	100	NA	0.25	100	NA
	AMP	≥32	17.6	82.4	≥32	23.9	76.1	≥32	18.4	81.6	≥32	15.0	85.0	≥32	16.2	83.8	≥32	17.0	83.0	≥32	18.3	81.7	≥32	18.8	81.2	≥32	17.7	82.3
	PEN	≥16	17.6	82.4	≥16	20.4	79.6	≥16	18.0	82.0	≥16	13.9	86.1	≥16	13.3	86.7	≥16	16.5	83.5	≥16	16.2	83.8	≥16	15.4	84.6	≥16	15.8	84.2
	AMC	≥16	59.3	40.7	≥16	66.4	33.6	≥16	73.7	26.3	≥16	79.7	20.3	≥16	74.2	25.8	≥16	75.4	24.6	≥16	64.7	35.3	≥16	68.4	31.6	≥16	71.3	28.7
	TZP	≥32	62.6	37.4	≥32	68.1	31.9	≥32	76.0	24.0	≥32	80.7	19.3	≥32	76.9	23.1	≥32	78.1	21.9	≥32	77.7	22.3	≥32	73.5	26.5	≥32	75.7	24.3
	CRO	≥128	56.0	39.6	≥128	64.6	32.7	≥128	69.1	22.1	≥128	76.5	18.7	≥128	66.8	21.3	≥128	69.6	21.0	≥128	65.5	20.9	≥128	67.9	25.6	≥128	67.7	23.3
	IPM	≥32	69.9	30.1	≥32	68.0	32.0	≥32	84.3	15.7	-	-	-	-	-	-	-	-	-	-	-	-	-	-	-	≥32	73.9	26.1
	MEM	-	-	-	≥32	76.9	23.1	≥32	76.6	21.9	≥32	83.4	15.0	≥32	83.8	14.1	≥32	83.0	12.9	16	83.1	11.2	≥32	75.2	20.5	≥32	81.3	15.4
	LVX	8	56.0	40.7	32	61.1	37.2	16	64.5	33.2	16	71.1	28.9	16	64.6	34.3	32	62.9	36.6	32	60.4	38.1	32	62.4	35.9	32	63.4	35.2
	LZD	4	100	0.0	4	100	0.0	4	100	0.0	4	100	0.0	4	100	0.0	4	100	0.0	2	100	0.0	2	100	0.0	4	100	0.0
	MIN	2	100	0.0	0.5	99.1	0.0	≤0.25	98.6	0.5	0.5	97.9	0.5	0.5	98.1	0.3	1	95.1	4.0	0.5	97.1	0.7	0.5	99.1	0.4	0.5	97.9	0.9
	VAN	2	100	0.0	1	100	0.0	1	100	0.0	1	100	0.0	1	100	0.0	1	100	0.0	1	100	0.0	1	100	0.0	1	100	0.0
*S. agalactiae*	TGC	0.12	100	NA	0.12	100	NA	0.12	100	NA	0.12	100	NA	0.12	100	NA	0.06	100	NA	0.25	100	NA	0.25	100	NA	0.12	100	NA
	AMP	0.12	100	NA	0.12	100	NA	0.12	100	NA	0.12	100	NA	0.12	100	NA	0.12	100	NA	0.12	100	NA	0.12	100	NA	0.12	100	NA
	PEN	≤0.06	100	NA	0.12	100	NA	0.12	100	NA	0.12	100	NA	0.12	100	NA	0.12	100	NA	≤0.06	100	NA	0.12	100	NA	0.12	100	NA
	AMC	0.06	NA	NA	0.12	NA	NA	0.12	NA	NA	0.12	NA	NA	0.06	NA	NA	0.12	NA	NA	0.12	NA	NA	0.12	NA	NA	0.12	NA	NA
	TZP	≤0.25	NA	NA	≤0.25	NA	NA	≤0.25	NA	NA	0.5	NA	NA	≤0.25	NA	NA	0.5	NA	NA	0.5	NA	NA	0.5	NA	NA	0.5	NA	NA
	CRO	0.06	100	NA	0.12	100	NA	0.12	100	NA	0.12	100	NA	0.06	100	NA	0.12	100	NA	0.12	100	NA	0.12	100	NA	0.12	100	NA
	IPM	0.25	NA	NA	≤0.12	NA	NA	≤0.12	NA	NA	-	-	-	-	-	-	-	-	-	-	-	-	-	-	-	0.25	NA	NA
	MEM	-	-	-	≤0.12	100	NA	≤0.12	100	NA	≤0.12	100	NA	≤0.12	100	NA	≤0.12	100	NA	≤0.12	100	NA	≤0.12	100	NA	≤0.12	100	NA
	LVX	1	100	0.0	1	100	0.0	1	98.9	0.0	1	97.2	1.4	1	99.2	0.8	1	97.4	2.6	1	100	0.0	1	98.3	1.7	1	98.9	0.8
	LZD	1	100	NA	1	100	NA	1	100	NA	1	100	NA	1	100	NA	1	100	NA	2	100	NA	2	100	NA	1	100	NA
	MIN	≥16	38.9	58.3	≥16	5.0	82.5	≥16	24.2	67.0	≥16	12.5	83.3	≥16	19.5	72.2	≥16	15.8	75.0	≥16	20.8	75.5	≥16	15.5	81.0	≥16	19.0	74.3
	VAN	0.5	100	NA	0.5	100	NA	0.5	100	NA	0.5	100	NA	0.5	100	NA	0.5	100	NA	1	100	NA	0.5	100	NA	0.5	100	NA
S. pneumoniae*	TGC	0.06	100	-	0.06	100	-	0.06	100	-	0.06	100	-	0.06	100	-	0.03	100	-	0.03	100	-	0.03	100	-	0.06	100	-
	AMP	≤0.06	-	-	0.5	-	-	0.12	-	-	0.12	-	-	1	-	-	2	-	-	4	-	-	2	-	-	0.5	-	-
	PEN	0.25	73.2	4.9	0.5	80.0	4.6	0.25	73.0	4.5	0.25	75.0	5.6	1	73.4	7.7	1	74.3	9.5	2	67.4	14.1	1	72.4	3.4	0.5	73.4	7.5
	AMC	0.06	100	0.0	0.5	98.5	0.0	0.12	99.1	0.0	0.06	97.6	2.4	0.5	96.5	2.1	1	96.2	1.9	2	91.3	3.3	1	96.6	0.0	0.5	96.7	1.5
	TZP	≤0.25	-	-	0.5	-	-	≤0.25	-	-	≤0.25	-	-	1	-	-	4	-	-	4	-	-	4	-	-	1	-	-
	CRO	0.12	97.6	2.4	0.25	100	0.0	0.12	98.2	0.0	0.5	100	0.0	0.5	94.4	2.8	1	93.3	1.0	1	92.4	0.0	0.5	100	0.0	0.5	96.3	0.9
	AZM	≥128	63.4	36.6	≥128	44.2	55.8	≥128	59.8	40.2	64	72.7	27.3	≥128	64.0	36.0	≥128	48.5	51.5	64	47.3	52.7	64	50.0	50.0	≥128	56.6	43.4
	CLR	≥128	63.4	36.6	≥128	44.2	55.8	≥128	59.8	39.2	64	74.5	25.5	≥128	64.0	35.2	≥128	48.5	51.5	64	48.4	51.6	64	50.0	50.0	≥128	57.0	42.7
	ERY	≥128	63.4	36.6	≥128	44.2	55.8	≥128	58.8	39.2	64	70.9	29.1	≥128	63.2	36.8	≥128	48.5	51.5	64	47.3	52.7	64	50.0	50.0	≥128	56.1	43.5
	CLI	≥128	68.3	31.7	≥128	65.1	34.9	≥128	76.3	23.7	≥128	80.0	20.0	≥128	67.2	32.8	≥128	60.4	38.6	≥128	59.3	40.7	≥128	57.1	42.9	≥128	67.0	32.9
	IPM	≤0.12	93.3	0.0	≤0.12	91.7	4.2	≤0.12	98.3	1.7	-	-	-	-	-	-	-	-	-	-	-	-	-	-	-	≤0.12	95.1	2.5
	MEM	0.5	88.5	3.8	1	82.4	11.8	0.25	92.3	5.8	≤0.12	92.9	4.8	0.25	90.2	4.9	0.5	89.5	5.7	0.5	87.0	6.5	0.5	89.7	3.4	0.5	89.8	5.5
	LVX	1	100	0.0	2	98.5	1.5	1	96.4	1.8	1	100	0.0	2	99.3	0.0	2	97.1	1.9	1	100	0.0	1	100	0.0	1	98.7	0.7
	LZD	1	100	-	1	100	-	1	100	-	1	100	-	1	100	-	1	100	-	1	100	-	1	100	-	1	100	-
	MIN	8	75.6	22.0	8	72.3	23.1	8	70.3	20.7	8	71.4	20.2	≥16	38.5	47.6	≥16	26.7	58.1	≥16	28.3	57.6	≥16	55.2	31.0	≥16	50.9	38.1
	VAN	0.5	100	-	0.5	100	-	0.5	100	-	0.5	100	-	0.5	100	-	0.5	100	-	0.5	100	-	0.5	100	-	0.5	100	-

^a^ AMC = amoxicillin-clavulanate; AMP = ampicillin; AZM = azithromycin; CRO = ceftriaxone; CLR = clarithromycin; CLI = clindamycin; ERY = erythromycin; IPM = imipenem; LVX = levofloxacin; LZD = linezolid; MEM = meropenem; MIN = minocycline; PEN = penicillin; TZP = piperacillin-tazobactam; TGC = tigecycline; VAN = vancomycin. NA = susceptibility or resistance breakpoint not available. - MIC90, %S and %R are not given where N ≤20. No imipenem data was collected after 2006. * Tetracycline breakpoints for *S. pneumoniae* are used here for minocycline. Susceptible (S), resistance (R) breakpoints: *Enterococcus* spp.: TGC, S ≤ 0.25; AMP, S ≤ 8, R ≥ 16; PEN, S ≤ 8, R ≥ 16; LVX, S ≤ 2, R ≥ 8; LZD, S ≤ 2, R ≥ 8; MIN, S ≤ 4, R ≥ 16; VAN, S ≤ 4, R ≥ 32; *S. aureus*: TGC, S ≤ 0.5; AMP, S ≤ 0.25, R ≥ 0.5; PEN, S ≤ 0.12, R ≥ 0.25; AMC, S ≤ 4, R ≥ 8; TZP, S ≤ 8, R ≥ 16; CRO, S ≤ 8, R ≥ 64; IPM, S ≤ 4, R ≥ 16; MEM, S ≤ 4, R ≥ 16; LVX, S ≤ 1, R ≥ 4; LZD, S ≤ 4, R ≥ 8; MIN, S ≤ 4, R ≥ 16; VAN, S ≤ 2, R ≥ 16; *S. agalactiae*: TGC, S ≤ 0.25; AMP, S ≤ 0.25; PEN, S ≤ 0.12; CRO, S ≤ 0.5; MEM, S ≤ 0.5; LVX, S ≤ 2, R ≥ 8; LZD, S ≤ 2; MIN, S ≤ 2, R ≥ 8; VAN, S ≤ 1; *S. pneumoniae*: TGC, S ≤ 0.06; PEN, S ≤ 0.06, R ≥ 2; AMC, S ≤ 2, R ≥ 8; CRO, S ≤ 1, R ≥ 4; AZM, S ≤ 0.5, R ≥ 2; CLR, S ≤ 0.25, R ≥ 1; ERY, S ≤ 0.25, R ≥ 1; CLI, S ≤ 0.25, R ≥ 1; IPM, S ≤ 0.12, R ≥ 1; MEM, S ≤ 0.25, R ≥ 1; LVX, S ≤ 2, R ≥ 8; LZD, S ≤ 2; MIN, S ≤ 2, R ≥ 8; VAN, S ≤ 1.

**Table 4:** Overall prevalence (N), susceptibility (%S) and resistance (%R) among multidrug-resistant isolates of *A. baumannii* and *P. aeruginosa* collected in Italy during T.E.S.T. 2004–2011.

Organism	Agent^a^	2004		2005		2006		2007		2008		2009		2010		2011		2004–2011	
		N = 13		N = 10 (8/2)		N = 59 (28/31)		N = 42		N = 117		N = 83		N = 104		N = 98		N = 526 (49/477)	
		%S	%R	%S	%R	%S	%R	%S	%R	%S	%R	%S	%R	%S	%R	%S	%R	%S	%R
*A. baumannii*	TZP	7.7	92.3	0.0	80.0	3.4	78.0	4.8	90.5	0.9	94.0	1.2	96.4	0.0	99.0	1.0	98.0	1.5	93.7
	CAZ	0.0	92.3	0.0	90.0	0.0	98.3	0.0	100	1.7	95.7	0.0	97.6	1.9	95.2	5.1	91.8	1.7	95.6
	CRO	0.0	100	0.0	90.0	0.0	98.3	0.0	100	0.0	100	0.0	100	1.0	98.1	3.1	89.8	0.8	97.3
	FEP	0.0	84.6	10.0	50.0	1.7	81.4	0.0	90.5	2.6	84.6	10.8	71.1	6.7	77.9	2.0	78.6	4.4	79.5
	IPM	46.2	53.8	-	-	50.0	32.1	-	-	-	-	-	-	-	-	-	-	49.0	38.8
	MEM	-	-	-	-	6.5	83.9	38.1	52.4	21.4	59.8	4.8	81.9	1.0	95.2	1.0	98.0	10.3	80.3
	LVX	0.0	100	0.0	100	0.0	93.2	0.0	95.2	0.0	98.3	0.0	97.6	0.0	100	0.0	100	0.0	98.1
	AMK	23.1	76.9	20.0	80.0	8.5	88.1	0.0	100	14.5	82.1	2.4	96.4	18.3	80.8	4.1	89.8	9.9	87.5
	MIN	100	0.0	100	0.0	94.9	1.7	100	0.0	82.1	3.4	57.8	10.8	37.5	10.6	62.2	2.0	69.4	5.1
**Organism**	**Agent^a^**	**2004**		**2005**		**2006**		**2007**		**2008**		**2009**		**2010**		**2011**		**2004–2011**	
		**N = 19 (17/2)**		**N = 13 (12/1)**		**N = 33 (19/14)**		**N = 20**		**N = 65**		**N = 43**		**N = 49**		**N = 38**		**N = 280 (48/232)**	
		**%S**	**%R**	**%S**	**%R**	**%S**	**%R**	**%S**	**%R**	**%S**	**%R**	**%S**	**%R**	**%S**	**%R**	**%S**	**%R**	**%S**	**%R**
*P. aeruginosa*	TZP	21.1	52.6	23.1	38.5	27.3	51.5	15.0	75.0	10.8	72.3	7.0	62.8	4.1	87.8	7.9	52.6	12.1	65.7
	CAZ	21.1	63.2	15.4	84.6	6.1	75.8	25.0	75.0	6.2	83.1	9.3	86.0	4.1	77.6	2.6	76.3	8.6	78.9
	FEP	0.0	73.7	15.4	53.8	3.0	81.8	25.0	55.0	9.2	70.8	14.0	67.4	8.2	51.0	0.0	86.8	8.6	68.6
	IPM	0.0	100	0.0	100	0.0	100	-	-	-	-	-	-	-	-	-	-	0.0	100
	MEM	-	-	-	-	0.0	100	0.0	100	1.5	96.9	2.3	95.3	0.0	95.9	0.0	97.4	0.9	97.0
	LVX	5.3	94.7	0.0	100	0.0	100	0.0	90.0	0.0	100	2.3	97.7	0.0	100	0.0	100	0.7	98.6
	AMK	78.9	10.5	38.5	38.5	66.7	21.2	65.0	20.0	53.8	21.5	55.8	32.6	65.3	26.5	39.5	44.7	57.5	27.1

^a^ AMK = amikacin; FEP = cefepime; CAZ = ceftazidime; CRO = ceftriaxone; IPM = imipenem; LVX = levofloxacin; MEM = meropenem; MIN = minocycline; TZP = piperacillin-tazobactam.. N = total number of isolates; numbers in parentheses represent total isolate number tested against imipenem and meropenem, respectively; where no parentheses are given, all isolates were tested against imipenem (2004–2006) or meropenem (2007–2011). - %S and %R are not given where N <10. No imipenem data was collected after 2006.

#### 3.1.3. *E.* *coli*

Amikacin, meropenem and tigecycline were all effective against *E. coli*, with >95% susceptibility during all years of surveillance (Table 2). Susceptibility of *E. coli* to cefepime was 93.4% in 2004, but this decreased to 77.4% in 2011, with some fluctuation in susceptibility during the years in between. *E. coli* were 28.4% susceptible to ampicillin and approximately 50% susceptible to levofloxacin from 2004–2011. The proportion of ESBL-producing *E. coli* in Italy was initially low from 2004 to 2007 (ranging from 12.6% in 2006 to 19.8% in 2004), but were notably higher between 2008 and 2011 (28.0–35.8%; Table 5). Tigecycline, meropenem and amikacin were highly active against ESBL-positive *E. coli* (100%, 98.8% and 94.2% susceptible, respectively) in this study (Table 6). *E. coli* were 99.5% susceptible to imipenem (Table 2) while 96.8% of ESBL-producing isolates were imipenem-susceptible (Table 6).

#### 3.1.4. *H.* *influenzae*

Isolates of *H. influenzae* were highly susceptible (>97.8% except for ampicillin [>87%] from 2004–2011) to all T.E.S.T. agents for which breakpoints were available (Table 2). Amikacin exhibited low activity against this pathogen (overall MIC_90_ 8 mg/L). The resistance of *H. influenzae* to ampicillin fluctuated during T.E.S.T., ranging from 2.6% in 2009 to 15.0% in 2010. The frequency of β-lactamase-producing *H. influenzae* peaked in Italy in 2005 (15.5%), and was lowest in 2009 (3.5%; Table 5), but there were no notable trends. All *H. influenzae* were imipenem-susceptible between 2004 and 2006 (Table 2).

#### 3.1.5. *K.* *oxytoca*

*K. oxytoca* were susceptible to most antimicrobials on the T.E.S.T. panel (Table 2). For all years combined, susceptibility was between 90%-100% for amikacin, cefepime, levofloxacin, meropenem and tigecycline; 80%-90% for ceftriaxone, minocycline and piperacillin-tazobactam; and was 77.1% for amoxicillin-clavulanate. The proportion of ESBL-producing *K. oxytoca* was highest in 2004 (12.5%) and 2008 (12.3%), but was low (<5%) in all other years, giving an overall value of 5.0% (Table 5). Tigecycline, amoxicillin-clavulanate, meropenem and amikacin maintained good activity (≥94.7% susceptibility) against ESBL-positive isolates of *K. oxytoca* (Table 6). In total, 98.8% of *K. oxytoca* isolates were susceptible to imipenem (2004–2006) (Table 2).

#### 3.1.6. *K.* *pneumoniae*

*K. pneumoniae* were less susceptible than *K. oxytoca* to most agents, apart from tigecycline for which 95.8% susceptibility was noted over all years (Table 2). From 2004 to 2011, *K. pneumoniae* were 80–90% susceptible to amikacin and meropenem, and 70–80% susceptible to cefepime, levofloxacin and piperacillin-tazobactam in Italy. The numbers of ESBL-producing *K. pneumoniae* ranged from 16.8% (2006) to 35.1% (2004), giving an overall value of 24.1% from 2004–2011 (Table 5). The most active agents against ESBL-positive *K. pneumoniae* were tigecycline (92.9% susceptible) and meropenem (81.2%) (Table 6). Among all *K. pneumoniae*, 95.6% of isolates were imipenem-susceptible between 2004 and 2006 (Table 2) while 86.2% of ESBL-positive isolates were susceptible (Table 6).

**Table 5:** Overall prevalence (N) and rate of resistant isolates (n, %) among Gram-negative and Gram-positive isolates collected in Italy during T.E.S.T. 2004–2011.

Organism	2004		2005		2006		2007		2008		2009		2010		2011		2004–2011	
	n/N	%	n/N	%	n/N	%	n/N	%	n/N	%	n/N	%	n/N	%	n/N	%	n/N	%
**Gram-negative**																		
ESBL-producing *E. coli*	18/91	19.8	18/139	12.9	32/254	12.6	28/190	14.7	121/432	28.0	76/252	30.2	102/285	35.8	84/288	29.2	479/1931	24.8
β-lactamase-producing *H. influenzae*	5/45	11.1	11/71	15.5	12/109	11.0	11/75	14.7	23/155	14.8	4/115	3.5	12/80	15.0	6/89	6.7	84/739	11.4
ESBL-producing *K. oxytoca*	2/16	12.5	0/29	0.0	2/67	3.0	1/46	2.2	9/73	12.3	3/69	4.3	2/41	4.9	0/39	0.0	19/380	5.0
ESBL-producing *K. pneumoniae*	27/77	35.1	18/86	20.9	32/191	16.8	33/127	26.0	82/329	24.9	42/200	21.0	73/225	32.4	29/160	18.1	336/1395	24.1
**Gram-positive**																		
Vancomycin-resistant *E. faecalis*	6/32	18.8	2/52	3.8	3/106	2.8	2/84	2.4	5/132	3.8	3/92	3.3	5/115	4.3	0/127	0.0	26/740	3.5
Vancomycin-resistant *E. faecium*	4/22	18.2	4/16	25.0	19/40	47.5	2/19	10.5	14/103	13.6	6/50	12.0	6/45	13.3	0/41	0.0	55/336	16.4
Methicillin-resistant *S. aureus*	41/91	45.1	43/113	38.1	72/217	33.2	52/187	27.8	128/376	34.0	66/224	29.5	103/278	37.1	77/234	32.9	582/1720	33.8
Penicillin-resistant *S. pneumoniae*	2/41	4.9	3/65	4.6	5/111	4.5	5/84	6.0	11/143	7.7	10/105	9.5	13/92	14.1	1/29	3.4	50/670	7.5

n = number of resistant isolates; N = total number of isolates. ESBL = extended-spectrum β-lactamase.

**Table 6:** MIC_90_ (mg/L), antimicrobial susceptibility (%S), and antimicrobial resistance (%R) for resistant Gram-negative and Gram-positive isolates collected in Italy during T.E.S.T. 2004–2011.

Organism	Agent^a^	2004			2005			2006			2007			2008			2009			2010			2011			2004–11		
		MIC_90_	%S	%R	MIC_90_	%S	%R	MIC_90_	%S	%R	MIC_90_	%S	%R	MIC_90_	%S	%R	MIC_90_	%S	%R	MIC_90_	%S	%R	MIC_90_	%S	%R	MIC_90_	%S	%R
Gram-negative																												
ESBL-positive*E. coli*	TGC	0.25	100	0.0	0.5	100	0.0	0.5	100	0.0	0.5	100	0.0	0.5	100	0.0	0.5	100	0.0	0.5	100	0.0	0.5	100	0.0	0.5	100	0.0
	AMP	≥64	0.0	100	≥64	0.0	100	≥64	0.0	100	≥64	0.0	100	≥64	0.0	100	≥64	0.0	100	≥64	0.0	100	≥64	0.0	100	≥64	0.0	100
	AMC	≥64	16.7	55.6	≥64	11.1	50.0	32	18.8	34.4	32	17.9	21.4	32	25.6	28.9	32	13.2	39.5	32	19.6	30.4	32	50.0	13.1	32	24.8	29.9
	TZP	≥256	61.1	27.8	≥256	66.7	22.2	64	71.9	6.3	64	67.9	7.1	128	66.9	15.7	≥256	53.9	17.1	128	68.6	11.8	64	79.8	8.3	128	67.6	13.4
	CRO	≥128	16.7	77.8	≥128	0.0	94.4	≥128	0.0	100	≥128	0.0	100	≥128	0.0	100	≥128	0.0	100	≥128	3.9	96.1	64	0.0	97.6	≥128	1.5	97.7
	FEP	≥64	66.7	22.2	≥64	16.7	72.2	≥64	18.8	59.4	≥64	14.3	75.0	≥64	26.4	59.5	≥64	23.7	72.4	≥64	26.5	54.9	≥64	34.5	47.6	≥64	27.3	58.5
	IPM	0.5	100	0.0	0.5	100	0.0	0.5	92.6	0.0	-	-	-	-	-	-	-	-	-	-	-	-	-	-	-	0.5	96.8	0.0
	MEM	-	-	-	-	-	-	-	-	-	0.12	100	0.0	0.12	96.7	0.8	≤0.06	98.7	0.0	≤0.06	100	0.0	≤0.06	100	0.0	≤0.06	98.8	0.2
	LVX	≥16	27.8	72.2	≥16	16.7	83.3	≥16	6.3	90.6	≥16	10.7	85.7	≥16	9.9	87.6	≥16	11.8	88.2	≥16	10.8	89.2	≥16	10.7	82.1	≥16	11.3	86.4
	AMK	16	100	0.0	32	88.9	5.6	16	93.8	3.1	16	92.9	3.6	16	90.1	5.0	16	93.4	2.6	16	96.1	1.0	16	98.8	0.0	16	94.2	2.5
	MIN	≥32	61.1	33.3	≥32	61.1	16.7	≥32	65.6	28.1	16	71.4	17.9	16	67.8	14.0	16	69.7	14.5	16	57.8	22.5	16	75.0	13.1	16	66.8	17.7
BL-pos *H. influenzae*	TGC	-	-	-	0.25	100	NA	0.25	100	NA	0.25	100	NA	0.25	100	NA	-	-	-	0.25	100	NA	-	-	-	0.25	100	NA
	AMP	-	-	-	≥64	9.1	90.9	32	0.0	83.3	≥64	0.0	100	≥64	0.0	95.7	-	-	-	≥64	0.0	100	-	-	-	≥64	1.2	92.9
	AMC	-	-	-	16	72.7	27.3	1	100	0.0	2	100	0.0	1	100	0.0	-	-	-	2	100	0.0	-	-	-	2	96.4	3.6
	TZP	-	-	-	1	90.9	9.1	0.25	100	0.0	≤0.06	100	0.0	≤0.06	100	0.0	-	-	-	≤0.06	100	0.0	-	-	-	≤0.06	98.8	1.2
	CRO	-	-	-	1	100	NA	0.12	100	NA	0.12	100	NA	≤0.06	100	NA	-	-	-	≤0.06	100	NA	-	-	-	≤0.06	100	NA
	FEP	-	-	-	1	100	NA	≤0.5	100	NA	≤0.5	90.9	NA	≤0.5	100	NA	-	-	-	≤0.5	100	NA	-	-	-	≤0.5	98.8	NA
	IPM	-	-	-	-	-	-	-	-	-	-	-	-	-	-	-	-	-	-	-	-	-	-	-	-	1	100	NA
	MEM	-	-	-	-	-	-	-	-	-	0.12	100	NA	0.12	100	NA	-	-	-	0.12	100	NA	-	-	-	0.12	100	NA
	LVX	-	-	-	0.12	100	NA	0.015	100	NA	0.03	100	NA	0.03	100	NA	-	-	-	0.03	100	NA	-	-	-	0.03	100	NA
	AMK	-	-	-	8	NA	NA	8	NA	NA	8	NA	NA	8	NA	NA	-	-	-	8	NA	NA	-	-	-	8	NA	NA
	MIN	-	-	-	2	90.9	0.0	≤0.5	100	0.0	2	100	0.0	1	100	0.0	-	-	-	1	100	0.0	-	-	-	1	98.8	0.0
ESBL-positive*K. oxytoca*	TGC	-	-	-	-	-	-	-	-	-	-	-	-	-	-	-	-	-	-	-	-	-	-	-	-	1	94.7	0.0
	AMP	-	-	-	-	-	-	-	-	-	-	-	-	-	-	-	-	-	-	-	-	-	-	-	-	≥64	0.0	100
	AMC	-	-	-	-	-	-	-	-	-	-	-	-	-	-	-	-	-	-	-	-	-	-	-	-	≥64	31.6	36.8
	TZP	-	-	-	-	-	-	-	-	-	-	-	-	-	-	-	-	-	-	-	-	-	-	-	-	≥256	47.4	47.4
	CRO	-	-	-	-	-	-	-	-	-	-	-	-	-	-	-	-	-	-	-	-	-	-	-	-	≥128	10.5	89.5
	FEP	-	-	-	-	-	-	-	-	-	-	-	-	-	-	-	-	-	-	-	-	-	-	-	-	≥64	73.7	15.8
	MEM	-	-	-	-	-	-	-	-	-	-	-	-	-	-	-	-	-	-	-	-	-	-	-	-	0.5	100	0.0
	LVX	-	-	-	-	-	-	-	-	-	-	-	-	-	-	-	-	-	-	-	-	-	-	-	-	≥16	52.6	36.8
	AMK	-	-	-	-	-	-	-	-	-	-	-	-	-	-	-	-	-	-	-	-	-	-	-	-	8	94.7	5.3
	MIN	-	-	-	-	-	-	-	-	-	-	-	-	-	-	-	-	-	-	-	-	-	-	-	-	≥32	68.4	10.5
ESBL-positive*K. pneumoniae*	TGC	2	96.3	0.0	2	100	0.0	4	84.4	6.3	2	100	0.0	4	86.6	6.1	2	97.6	2.4	2	91.8	0.0	2	100	0.0	2	92.9	2.4
	AMP	≥64	0.0	100	≥64	0.0	100	≥64	0.0	100	≥64	0.0	100	≥64	0.0	98.8	≥64	0.0	100	≥64	1.4	97.3	≥64	0.0	100	≥64	0.3	99.1
	AMC	≥64	37.0	29.6	≥64	27.8	44.4	≥64	12.5	59.4	32	15.2	45.5	32	23.2	40.2	≥64	14.3	61.9	≥64	16.4	50.7	≥64	34.5	31.0	≥64	21.1	46.1
	TZP	≥256	51.9	33.3	128	38.9	16.7	≥256	28.1	62.5	≥256	33.3	57.6	≥256	39.0	45.1	≥256	21.4	59.5	≥256	32.9	50.7	≥256	58.6	31.0	≥256	36.6	47.3
	CRO	≥128	3.7	96.3	≥128	0.0	94.4	≥128	3.1	90.6	≥128	0.0	100	≥128	0.0	100	≥128	0.0	100	≥128	8.2	91.8	64	3.4	93.1	≥128	2.7	96.1
	FEP	≥64	66.7	22.2	≥64	50.0	33.3	≥64	31.3	50.0	≥64	27.3	63.6	≥64	26.8	68.3	≥64	21.4	71.4	≥64	27.4	69.9	≥64	55.2	31.0	≥64	33.6	58.0
	IPM	0.5	100	0.0	0.5	100	0.0	2	55.0	0.0	-	-	-	-	-	-	-	-	-	-	-	-	-	-	-	2	86.2	0.0
	MEM	-	-	-	-	-	-	0.25	100	0.0	2	60.6	6.1	2	82.9	4.9	4	69.0	11.9	2	87.7	2.7	1	93.1	3.4	2	81.2	5.2
	LVX	≥16	55.6	25.9	≥16	44.4	50.0	≥16	40.6	59.4	≥16	30.3	66.7	≥16	26.8	67.1	≥16	16.7	81.0	≥16	31.5	65.8	≥16	37.9	55.2	≥16	32.4	62.5
	AMK	32	81.5	0.0	≥128	72.2	27.8	≥128	68.8	31.3	≥128	72.7	27.3	≥128	69.5	29.3	≥128	71.4	23.8	≥128	78.1	20.5	16	93.1	3.4	≥128	75.0	22.0
	MIN	16	59.3	25.9	≥32	50.0	38.9	≥32	34.4	59.4	≥32	60.6	33.3	≥32	36.6	56.1	≥32	45.2	35.7	≥32	30.1	49.3	≥32	69.0	17.2	≥32	43.8	43.5
VR *E. faecalis*	TGC	-	-	-	-	-	-	-	-	-	-	-	-	-	-	-	-	-	-	-	-	-	-	-	-	0.25	100	NA
	AMP	-	-	-	-	-	-	-	-	-	-	-	-	-	-	-	-	-	-	-	-	-	-	-	-	4	100	0.0
	PEN	-	-	-	-	-	-	-	-	-	-	-	-	-	-	-	-	-	-	-	-	-	-	-	-	8	100	0.0
	AMC	-	-	-	-	-	-	-	-	-	-	-	-	-	-	-	-	-	-	-	-	-	-	-	-	2	NA	NA
	TZP	-	-	-	-	-	-	-	-	-	-	-	-	-	-	-	-	-	-	-	-	-	-	-	-	16	NA	NA
	CRO	-	-	-	-	-	-	-	-	-	-	-	-	-	-	-	-	-	-	-	-	-	-	-	-	≥128	NA	NA
	MEM	-	-	-	-	-	-	-	-	-	-	-	-	-	-	-	-	-	-	-	-	-	-	-	-	16	NA	NA
	LVX	-	-	-	-	-	-	-	-	-	-	-	-	-	-	-	-	-	-	-	-	-	-	-	-	≥64	0.0	92.3
	MIN	-	-	-	-	-	-	-	-	-	-	-	-	-	-	-	-	-	-	-	-	-	-	-	-	≥16	19.2	30.8
	LZD	-	-	-	-	-	-	-	-	-	-	-	-	-	-	-	-	-	-	-	-	-	-	-	-	2	100	0.0
VR *E. faecium*	TGC	-	-	-	-	-	-	0.12	100	NA	-	-	-	1	78.6	NA	-	-	-	-	-	-	-	-	-	0.12	94.5	NA
	AMP	-	-	-	-	-	-	≥32	0.0	100	-	-	-	≥32	0.0	100	-	-	-	-	-	-	-	-	-	≥32	5.5	94.5
	PEN	-	-	-	-	-	-	≥16	0.0	100	-	-	-	≥16	7.1	92.9	-	-	-	-	-	-	-	-	-	≥16	5.5	94.5
	AMC	-	-	-	-	-	-	≥16	NA	NA	-	-	-	≥16	NA	NA	-	-	-	-	-	-	-	-	-	≥16	NA	NA
	TZP	-	-	-	-	-	-	≥32	NA	NA	-	-	-	≥32	NA	NA	-	-	-	-	-	-	-	-	-	≥32	NA	NA
	CRO	-	-	-	-	-	-	≥128	NA	NA	-	-	-	≥128	NA	NA	-	-	-	-	-	-	-	-	-	≥128	NA	NA
	IPM	-	-	-	-	-	-	-	-	-	-	-	-	-	-	-	-	-	-	-	-	-	-	-	-	≥32	NA	NA
	MEM	-	-	-	-	-	-	≥32	NA	NA	-	-	-	≥32	NA	NA	-	-	-	-	-	-	-	-	-	≥32	NA	NA
	LVX	-	-	-	-	-	-	≥64	0.0	100	-	-	-	≥64	0.0	100	-	-	-	-	-	-	-	-	-	≥64	1.8	98.2
	MIN	-	-	-	-	-	-	≥16	84.2	10.5	-	-	-	≥16	71.4	21.4	-	-	-	-	-	-	-	-	-	≥16	70.9	21.8
	LZD	-	-	-	-	-	-	2	100	0.0	-	-	-	2	92.9	7.1	-	-	-	-	-	-	-	-	-	2	96.4	1.8
MRSA	TGC	0.25	100	NA	0.12	100	NA	0.25	100	NA	0.25	100	NA	0.25	100	NA	0.5	100	NA	0.25	100	NA	0.5	100	NA	0.25	100	NA
	AMP	≥32	0.0	100	≥32	0.0	100	≥32	0.0	100	≥32	0.0	100	≥32	0.0	100	≥32	0.0	100	≥32	0.0	100	≥32	0.0	100	≥32	0.0	100
	PEN	≥16	0.0	100	≥16	0.0	100	≥16	0.0	100	≥16	0.0	100	≥16	0.0	100	≥16	0.0	100	≥16	0.0	100	≥16	0.0	100	≥16	0.0	100
	AMC	≥16	9.8	90.2	≥16	11.6	88.4	≥16	20.8	79.2	≥16	26.9	73.1	≥16	24.2	75.8	≥16	16.7	83.3	≥16	4.9	95.1	≥16	3.9	96.1	≥16	15.1	84.9
	TZP	≥32	17.1	82.9	≥32	16.3	83.7	≥32	27.8	72.2	≥32	30.8	69.2	≥32	32.0	68.0	≥32	25.8	74.2	≥32	39.8	60.2	≥32	19.5	80.5	≥32	28.2	71.8
	CRO	≥128	4.9	87.8	≥128	7.0	86.0	≥128	6.9	66.7	≥128	17.3	67.3	≥128	5.5	62.5	≥128	6.1	71.2	≥128	9.7	56.3	≥128	5.2	77.9	≥128	7.6	68.9
	IPM	≥32	32.4	67.6	≥32	8.6	91.4	≥32	26.3	73.7	-	-	-	-	-	-	-	-	-	-	-	-	-	-	-	≥32	22.0	78.0
	MEM	-	-	-	-	-	-	≥32	43.4	52.8	≥32	40.4	53.8	≥32	52.3	41.4	≥32	42.4	43.9	≥32	54.4	30.1	≥32	24.7	62.3	≥32	44.8	45.4
	LVX	16	12.2	85.4	≥64	4.7	93.0	32	1.4	97.2	32	3.8	96.2	≥64	11.7	87.5	32	4.5	95.5	≥64	5.8	92.2	≥64	5.2	93.5	≥64	6.5	92.3
	MIN	4	100	0.0	≤0.25	100	0.0	1	97.2	1.4	0.5	94.2	1.9	2	96.9	0.0	2	97.0	1.5	1	96.1	0.0	2	97.4	1.3	2	97.1	0.7
	LZD	4	100	0.0	2	100	0.0	4	100	0.0	2	100	0.0	2	100	0.0	2	100	0.0	2	100	0.0	2	100	0.0	2	100	0.0
	VAN	2	100	0.0	1	100	0.0	2	100	0.0	1	100	0.0	1	100	0.0	1	100	0.0	2	100	0.0	1	100	0.0	2	100	0.0
Pen-R *S. pneumoniae*	TGC	-	-	-	-	-	-	-	-	-	-	-	-	0.03	100	NA	0.03	100	NA	0.03	100	NA	-	-	-	0.03	100	NA
	AMP	-	-	-	-	-	-	-	-	-	-	-	-	8	NA	NA	8	NA	NA	16	NA	NA	-	-	-	8	NA	NA
	AMC	-	-	-	-	-	-	-	-	-	-	-	-	8	54.5	27.3	8	60.0	20.0	8	46.2	23.1	-	-	-	8	60.0	20.0
	TZP	-	-	-	-	-	-	-	-	-	-	-	-	8	NA	NA	8	NA	NA	8	NA	NA	-	-	-	8	NA	NA
	AZM	-	-	-	-	-	-	-	-	-	-	-	-	≥128	18.2	81.8	64	30.0	70.0	≥128	23.1	76.9	-	-	-	≥128	34.7	65.3
	CLR	-	-	-	-	-	-	-	-	-	-	-	-	≥128	18.2	81.8	64	30.0	70.0	≥128	23.1	76.9	-	-	-	≥128	34.7	65.3
	ERY	-	-	-	-	-	-	-	-	-	-	-	-	≥128	18.2	81.8	64	30.0	70.0	≥128	23.1	76.9	-	-	-	≥128	34.7	65.3
	CLI	-	-	-	-	-	-	-	-	-	-	-	-	≥128	18.2	81.8	64	60.0	30.0	≥128	46.2	53.8	-	-	-	≥128	49.0	49.0
	CRO	-	-	-	-	-	-	-	-	-	-	-	-	8	36.4	27.3	2	30.0	10.0	2	53.8	0.0	-	-	-	2	54.0	10.0
	MEM	-	-	-	-	-	-	-	-	-	-	-	-	1	0.0	63.6	1	0.0	60.0	1	15.4	46.2	-	-	-	1	4.2	62.5
	LVX	-	-	-	-	-	-	-	-	-	-	-	-	2	90.9	0.0	1	100	0.0	1	100	0.0	-	-	-	2	96.0	2.0
	MIN	-	-	-	-	-	-	-	-	-	-	-	-	≥16	9.0	54.5	8	70.0	30.0	≥16	23.1	76.9	-	-	-	≥16	46.0	42.0
	LZD	-	-	-	-	-	-	-	-	-	-	-	-	1	100	NA	1	100	NA	1	100	NA	-	-	-	1	100	NA
	VAN	-	-	-	-	-	-	-	-	-	-	-	-	0.5	100	NA	1	100	NA	0.5	100	NA	-	-	-	1	100	NA

^a^ AMC = amoxicillin-clavulanate; AMP = ampicillin; AZM = azithromycin; CRO = ceftriaxone; CLR = clarithromycin; CLI = clindamycin; ERY = erythromycin; IPM = imipenem; LVX = levofloxacin; LZD = linezolid; MEM = meropenem; MIN = minocycline; PEN = penicillin; TZP = piperacillin-tazobactam; TGC = tigecycline; VAN = vancomycin. NA = susceptibility or resistance breakpoint not available. - MIC90, %S and %R are not given where N ≤10. No imipenem data was collected after 2006.

#### 3.1.7. *P.* *aeruginosa*

Where breakpoints were available, *P. aeruginosa* had lower susceptibility than other Gram-negative pathogens to most T.E.S.T. agents (Table 2). This organism was most susceptible to amikacin (88.2% from 2004–2011); all other antimicrobial susceptibilities were between 54.6% (levofloxacin) and 64.1% (cefepime). *P. aeruginosa* isolates showed the highest levels of resistance to levofloxacin (39.1%) and the lowest levels to amikacin (6.6%) over all T.E.S.T. years. Breakpoints were not available for tigecycline and the activity of this agent was low against *P. aeruginosa* (MIC_90_ 16mg/L for all years combined). Two hundred and eighty MDR *P. aeruginosa* isolates were collected in Italy over all years of surveillance (19.1% of all *P. aeruginosa* isolates; Table 4). MDR *P. aeruginosa* showed high resistance (≥65.7%) to cefepime, ceftazidime, levofloxacin, meropenem and piperacillin-tazobactam; the lowest level of resistance was for amikacin (27.1%). A resistance rate of 25.1% to imipenem was reported among all *P. aeruginosa*; 100% of MDR *P. aeruginosa* isolates were resistant to imipenem (Table 4).

#### 3.1.8. *S.* *marcescens*

The most effective antimicrobials against this organism were amikacin, cefepime, levofloxacin, meropenem, piperacillin-tazobactam and tigecycline (>90% mean susceptibility over 2004–2011; Table 2). Of these agents, isolates of *S. marcescens* were least resistant to amikacin and tigecycline (<1% overall resistance). Ceftriaxone susceptibility was lowest in 2004 (64.3%) and peaked at 94.0% in 2007. Imipenem susceptibility was observed among 85.7% of *S. marcescens* isolates (Table 2).

### 3.2. Gram-Positive Isolates

Over all T.E.S.T. years in Italy, the Gram-positive organisms collected, in order of highest to lowest importance, were *S. aureus* (42.1%), *E. faecalis* (18.1%), *S. pneumoniae* (16.5%), *S. agalactiae* (15.0%) and *E. faecium* (8.2%; Table 1). Generally speaking, tigecycline, linezolid, vancomycin and the carbapenems were active against the majority of Gram-positive isolates in this study.

#### 3.2.1. *E.* *faecalis*

Over all T.E.S.T. years, isolates were >90% susceptible to ampicillin, linezolid, penicillin, tigecycline and vancomycin, and least susceptible to minocycline (25.3%; Table 3). The highest resistance from 2004–2011 was to minocycline (38.5%) and levofloxacin (33.2%). No linezolid-resistant isolates were collected over the study period. Only 0.4% of isolates were resistant to ampicillin or penicillin; all these resistant isolates were collected in 2010. Vancomycin-resistant *E. faecalis* were most prevalent in 2004 (18.8%), after which time the proportions of resistant isolates decreased, down to 0% in 2011 (Table 5). All vancomycin-resistant *E. faecalis* isolates were susceptible to tigecycline, ampicillin, penicillin and linezolid (Table 6).

#### 3.2.2. *E.* *faecium*

During the T.E.S.T. study, the most effective agents against this pathogen were linezolid and tigecycline (>98% susceptibility; Table 3). Two linezolid-resistant isolates were collected in 2008 (1.9%), resulting in a 0.6% resistance rate over the total study period. Overall, *E. faecium* isolates from Italy were least susceptible (12.5%) and most resistant (83.0%) to levofloxacin. Resistance to ampicillin, penicillin and vancomycin increased from 2004 to 2006 to a maximum of 90.0%, 92.5% and 47.5%, respectively. From 2006 onwards, however, resistance to these three antimicrobials decreased, giving overall resistance values of 79.8%, 70.5% and 16.4%, respectively. The frequency of vancomycin-resistant isolates of *E. faecium* increased from 18.2% in 2004 to a maximum of 47.5% in 2006 but then decreased to 0.0% in 2011 (Table 5). High susceptibility to linezolid (96.4%) and tigecycline (94.5%) was noted among vancomycin-resistant *E. faecium* isolates (Table 6).

#### 3.2.3. *S.* *agalactiae*

*S. agalactiae* were >97% susceptible to most agents (ampicillin, ceftriaxone, levofloxacin, linezolid, meropenem, penicillin, tigecycline and vancomycin) in every T.E.S.T. year (Table 3). Low susceptibility and high resistance were reported only to minocycline (19.0% and 74.3%, respectively).

#### 3.2.4. *S.* *aureus*

*S. aureus* were 100% susceptible to linezolid, tigecycline and vancomycin in every year of surveillance in Italy (Table 3). No isolates were resistant to linezolid or vancomycin throughout T.E.S.T. *S. aureus* were 81.3% and 97.9% susceptible to meropenem and minocycline, respectively, from 2004–2011, but whereas overall resistance to minocycline was <1%, meropenem resistance was 15.4%. This pathogen only had high resistance to ampicillin and penicillin (82.3% and 84.2% resistance, respectively, for all study years combined). Methicillin resistance was noted in 33.8% of isolates overall, with the annual frequencies of MRSA ranging from 27.8% in 2007 to 45.1% in 2004 (Table 5). All MRSA isolates were susceptible to tigecycline, linezolid and vancomycin, while 97.1% were susceptible to minocycline (Table 6).

#### 3.2.5. *S.* *pneumoniae*

From 2004–2011, all isolates were susceptible to linezolid, tigecycline and vancomycin, and >95% susceptibility was noted for ceftriaxone and levofloxacin (Table 3). *S. pneumoniae* were ≤5% resistant to all of the above agents, with the exception of meropenem (3.4%-11.8% resistance annually). Susceptibility to minocycline decreased from approximately 70–75% in 2004–2007 to between 26.7% and 55.2% annually over the period 2008 to 2011. Penicillin susceptibility ranged from 67.4% to 80.0% between 2004 and 2011. Azithromycin, clarithromycin and erythromycin had similar overall susceptibility (56–57%) and resistance (42.5–43.5%) values for *S. pneumoniae* (Table 3). *S. pneumoniae* was more susceptible (67%) and less resistant (32.9%) to clindamycin than to the macrolides between 2004–2011. Penicillin-resistant *S. pneumoniae* (PRSP) increased in prevalence from 4.9% in 2004 to 14.1% in 2010, but decreased to 3.4% in 2011 (Table 5). All PRSP isolates were tigecycline-, linezolid- and vancomycin-susceptible, while 96.0% were susceptible to levofloxacin (Table 6). Imipenem susceptibility was noted among 95.1% of *S. pneumoniae* isolates (2004–2006) (Table 3).

## 4. Discussion

Tigecycline is licensed in Italy to treat complicated intra-abdominal and skin and soft tissue infections. Good clinical results for tigecycline have been demonstrated previously: for example, Bassetti *et al.* [11] showed a 76.5% (13/17) success rate for tigecycline in the treatment of complicated skin and soft tissue infections and an 82.8% (72/87) success rate against peritonitis. This clinical success is reflected by high tigecycline susceptibility for most pathogens over the course of the T.E.S.T. study between 2004 and 2011: Gram-negative pathogens showed >94% tigecycline susceptibility while Gram-positives were >98% susceptible in the present report.

The two Gram-negative organisms in this study for which tigecycline breakpoints were not available were *A. baumannii* and *P. aeruginosa*. Against *A. baumannii*, tigecycline had the lowest overall MIC_90_ (2 mg/L) of all T.E.S.T. agents in Italy. A previously published Italian surveillance study comprising nine hospitals also determined an MIC_90_ of 2 mg/L for tigecycline against *A. baumannii* isolates collected between 2003 and 2004 [12]. As in the current analysis, the one-year study found that *A. baumannii* isolates had low (≤51%) susceptibility to amikacin, cefepime, ceftazidime, meropenem and piperacillin-tazobactam. The proportion of *A. baumannii* resistant to piperacillin-tazobactam in the 2003–2004 study (44%) was lower than to the overall value in this T.E.S.T. paper (63.3%; 2004–2011). However, the 2003–2004 frequency of imipenem-resistant isolates (50%) was around 36% higher than T.E.S.T. (13.7%; 2004–2006). Both studies used CLSI methodologies to determine imipenem susceptibility, but there were 27 countrywide centres collecting isolates for T.E.S.T., compared with nine centres in the central-south region of Italy for the 2003–2004 study. The high incidence of imipenem resistance in the one-year study could, therefore, be due to a localised outbreak of imipenem-resistant *A. baumannii* infections in this region of Italy during 2003–2004. *A. baumannii* gained resistance to most antimicrobials on the panel over the course of the T.E.S.T. study in Italy as described in this report, and the greatest increase in resistance was to piperacillin-tazobactam (49.1% from 2004 to 2011). There were also >40% increases in resistance to amikacin and levofloxacin.

Epidemiological studies of *A. baumannii* in Italy have focussed mostly on carbapenem-resistant or MDR isolates. Lambiase *et al.* [13] examined 567 *A. baumannii* isolates from an ICU in Naples between 2007 and 2010, and found that all isolates were MDR, including carbapenems; these isolates were clonal in nature, all possessing the *bla*_OXA-51-like_ and *bla*_OXA-58-like_ genes. D’Arezzo *et al.* [14] reported a high (60.5%) prevalence of elevated (MIC≥128 mg/L) resistance to imipenem among 111 *A. baumannii* isolates, associated with *bla*_OXA-58-like_ (22.8%) or *bla*_OXA-51-like_ (71.1%) genes. Most of these isolates (95.6%) were related to international clonal lineage II. In a study of six hospitals in Florence, Donnarumma *et al.* [15] showed three main clonal groups of *A. baumannii*, A1, A2 and A3; A1 was genetically related to the European EU II clone. All isolates possesses the *bla*_OXA-51-like_ gene, and 65% of these isolates were resistant to imipenem. As reported globally [16], clonal lineages appear to predominate among *A. baumannii* isolates in Italy, although there may be variation between different geographical areas.

In the 2003–2004 Italian study by Mezzatesta *et al.* [12], 49.5% of the 107 *A. baumannii* isolates tested were resistant to three antimicrobial classes (specifically fluoroquinolones, ceftazidime, and aminoglycosides or imipenem). This was only slightly lower than the proportion of MDR *A. baumannii* collected over the T.E.S.T. surveillance period in Italy (60%). In a separate study of seven centres in Rome carried out from 2004 to 2005, antimicrobial susceptibility was determined for 22 MDR *A. baumannii* patient isolates [17]. Of these 22 isolates, 21 (95.5%) were resistant to levofloxacin, 19 (86.4%) were resistant to piperacillin-tazobactam, and 14 (63.6%) were resistant to amikacin. The MDR *A. baumannii* isolates collected in T.E.S.T. were highly resistant to levofloxacin (98.1%), piperacillin-tazobactam (93.7%) and amikacin (87.5%) over all years of surveillance. In the seven-centre study by Principe *et al.* [17], three MDR *A. baumannii* isolates (13.6%) were resistant to tigecycline (using the FDA Enterobacteriaceae breakpoints for tigecycline [S, ≤2 mg/L; R, ≥8 mg/L]). A single Italian hospital also determined drug resistance among 50 MDR *A. baumannii* isolates collected between 2008 and 2009 [18]. Only 4% of these *A. baumannii* isolates were resistant to tigecycline (S, ≤2 mg/L; R, ≥8 mg/L). All isolates, however, were 100% resistant to imipenem, levofloxacin and piperacillin-tazobactam, and more than 90% resistant to amikacin, cefepime and ceftazidime. In this T.E.S.T. study, MDR *A. baumannii* isolates were highly resistant (>79%) to the same antimicrobials, excluding imipenem (38.8%) and minocycline (5.1%).

Compared with *A. baumannii*, a lower overall proportion of MDR *P. aeruginosa* (19.1%) was recorded in Italy during T.E.S.T. In 2010, a similar percentage of invasive *P. aeruginosa* isolates from Italy (20.8%) was reported to have resistance to three or more antibiotic classes among aminoglycosides, carbapenems, ceftazidime, fluoroquinolones, and piperacillin-tazobactam [19]. The individual resistance values for ceftazidime and piperacillin-tazobactam against these invasive isolates were 21.2% and 17.7%, respectively, slightly lower than the 2010 T.E.S.T. results in Italy (32.9% resistance for both agents).

The highest proportion of resistant Gram-negative pathogens in the current Italian study was observed among ESBL-producing *E. coli* (24.8% from 2004–2011). This is a dramatic increase compared to the 10.8% occurrence of ESBL-positive *E. coli* isolates nationally in 1999 [20]. An Italian single-hospital surveillance report from 2004 to 2007 identified 23.5% of *E. coli* as ESBL producers [21]. A more recent (2009–2010) study of another Italian hospital found that the most frequently observed multidrug-resistant pathogen was ESBL-producing *E. coli* (18.6% of all multidrug-resistant isolates) [22]. These data are in line with the findings from the current T.E.S.T. manuscript.

ESBL production has been linked with third-generation cephalosporin resistance [23]. The T.E.S.T. data showed that the resistance of *E. coli* to ceftriaxone in Italy increased more than two-fold between 2007 and 2008, during which time the prevalence of ESBL-producing *E. coli* isolates almost doubled. In 2010, the ECDC annual report found that in one Italian centre, all 23 of the invasive *E. coli* isolates resistant to third-generation cephalosporins were ESBL-producers [19]. In a 2007–2008 single hospital study of 13 countries, including Italy, higher mortality rates and longer hospital stays were associated with third-generation cephalosporin-resistant *E. coli* bloodstream infections [24]. These findings suggest that *E. coli* resistance in Italy is increasing, possibly due to the spread of ESBL-positive strains.

High proportions of ESBL-producing *K. pneumoniae* were also measured during T.E.S.T. (24.1% over all years). ESBL production has been associated with reduced carbapenem susceptibility in *K. pneumoniae* due to a loss of bacterial membrane permeability in some ESBL-producing isolates [25]. The recent ECDC report noted an increase in carbapenem-resistant *K. pneumoniae*, from 1% in 2006 to 27% in 2011 [1]. Another recent Italian paper has also highlighted a rise in carbapenem (imipenem and/or meropenem) non-susceptibility (intermediate plus resistant isolates) in *K. pneumoniae*, from 2.2% to 19.4% between 2009 and 2012, respectively [26]. These results are supported by the current T.E.S.T. study, in which *K. pneumoniae* resistance to meropenem increased in Italy from 1.4% in 2006 to 14.4% in 2011.

Although Gram-positive organisms were wholly susceptible to tigecycline, certain pathogens had low susceptibility to other antimicrobials during this T.E.S.T. study. *E. faecium*, for example, showed 79.8% resistance to ampicillin over all years of Italian surveillance. Similarly, an earlier report documented 70% ampicillin resistance among 913 *E. faecium* isolates from 20 Italian centres between 1993 and 1995 [27]. The more recent report from a single Italian teaching hospital by Manfredi and Nanetti [21] also found that ampicillin had limited activity against *E. faecium* (7.5%-18.5% susceptibility in 175 isolates from 2004–2007). Vancomycin resistance in *E. faecium*, however, appears to be declining in Italy, as the ECDC reported 21% vancomycin-resistant isolates in 2004 but 4% in 2010 [19]. This T.E.S.T. study showed similar results in 2004 (18.2% vancomycin resistance), decreasing to 13.3% resistance in 2010, before a further decline to 0% in 2011. This reduction in vancomycin-resistant *E. faecium* may be related to increased use of infection-control strategies in hospitals, which have been shown to reduce the incidence of vancomycin-resistant enterococci [28].

The current T.E.S.T. data showed that 33.8% of *S. aureus* isolates in Italy were resistant to methicillin, increasing in prevalence between 2007 (27.8%) and 2011 (32.9%) (although an overall decrease was noted compared to 2004 [45.1%]). Similarly, the ECDC reported a small increase in the proportion of MRSA between 2007 and 2010, from 33% to 37% [19]. The distribution of MRSA across Italy is complicated. In Torino, characterization of 90 MRSA isolates revealed that most belonged to SCCmec types I and II [29], while in the Emilia-Romagna region, 63% of MRSA clones showed spa-types t008 or t041 [30]. An examination of 10 Panton-Valentine leukocidin-positive MRSA isolates from Bolzano province revealed a heterogeneous sample, with eight different ST clonal types identified [31]. Italy thus possesses a polyclonal population of MRSA, with different clonal types occurring in separate regions in differing proportions. Also, there has been a blurring of the distinction between community-acquired and hospital- acquired MRSA: one recent report has shown a high prevalence of the USA-300 clone in central Italy among both community- and hospital-acquired isolates of MRSA [32].

Two key antimicrobials in the treatment of infections caused by MRSA are vancomycin and linezolid. There have been numerous reports of increasing non-susceptibility to vancomycin among MRSA isolates in recent years [33]. However, there is no evidence of this in the current study in Italy: vancomycin retains 100% activity against *S. aureus* (and MRSA) through the T.E.S.T. study in 2004–2011. Vancomycin “MIC creep” appears to be a regional occurrence and not yet a generalized trend; medical institutions should thus regularly monitor local vancomycin susceptibility among MRSA isolates [34]. Similarly, linezolid has retained its normal good activity against *S. aureus* (and MRSA) in the current study, with 100% susceptibility reported over all study years. Linezolid has previously been shown to be active against vancomycin-intermediate isolates of MRSA collected in Italy, so is an important clinical tool in the fight against resistant infections [35].

Some important differences are observed when resistance rates in Italy are compared to global rates, as reported in Pfizer’s online T.E.S.T. database [36]. In general, resistance levels to β-lactams (particularly ceftriaxone) and levofloxacin appear to be higher in Italy than globally. *A. baumannii* resistance to most antimicrobial agents is higher in Italy by approximately 15%, with the exceptions of amikacin and levofloxacin (resistance in Italy is > 20% higher) as well as imipenem and minocycline (resistance is similar). *E. coli* resistance is also high in Italy: ampicillin and cefepime resistance are around 7% higher, while ceftriaxone and levofloxacin resistance are approximately 12% higher than globally. Among *Enterobacter* spp., resistance was roughly 10% higher in Italy to ceftriaxone, levofloxacin and piperacillin-tazobactam. Amoxicillin-clavulanate, cefepime, ceftriaxone, levofloxacin and piperacillin-tazobactam resistance among Italian isolates of *K. pneumoniae* are approximately 6–8% higher than isolates globally. Resistance is roughly 6–10% higher among isolates of *P. aeruginosa* from Italy to all antimicrobial agents (excluding amikacin, to which resistance is the same in Italy as globally). Among *S. pneumoniae*, macrolide, minocycline and clindamycin resistance are about 10%, 12% and 14% higher in Italy, respectively, while penicillin resistance is approximately 7% lower than the global average. Implementation of and strict adherence to resistance control measures, such as ongoing resistance surveillance, improved hand hygiene/increased glove use and/or the use of antimicrobial stewardship programs [37,38], would almost certainly help to reduce the high levels of resistance observed in Italy.

The T.E.S.T. study, like all surveillance studies, suffers from inherent limitations. Although several centres participated in the T.E.S.T. study in Italy over 2004–2011, some contributed isolates over several years while others participated in a few years or even just one, causing fluctuations in isolate contribution both geographically and over time. Thus, regional variations in resistance in a given study year may have had a disproportionate influence on apparent national resistance levels.

Over all T.E.S.T. years, rates of β-lactamase-producing *H. influenzae*, ESBL-producing *K. oxytoca*, vancomycin-resistant *E. faecalis* and PRSP were ≤11.4% in Italy. Another encouraging result of this study was the identification of no vancomycin-resistant *Enterococci* in Italy in 2011. The above findings may indicate that these drug-resistant organisms are becoming less prevalent in Italian hospitals, and therefore, less of a threat to the welfare of patients. Other pathogens in Italy, including *E. coli* and *K. pneumoniae*, have shown increased resistance in recent years, due largely to the spread of ESBL-positive strains. Thus, these organisms must continue to be monitored for further changes in susceptibility in the future. The results of surveillance studies such as T.E.S.T. help members of the healthcare industry to monitor rates of *in vitro* susceptibility among important pathogens to widely used antimicrobial agents, both globally and regionally.

## 5. Conclusions

Tigecycline and linezolid exhibited very good activity against Gram-positive pathogens in Italy, with MIC_90_s ranging from 0.06 to 0.25 mg/L and 1–4 mg/L, respectively. Vancomycin and the carbapenems also showed good activity against select Gram-positive pathogens. Tigecycline was the most active agent against Gram-negative pathogens (with the exception of *P. aeruginosa*), with MIC_90_s between 0.25 and 2 mg/L (but 16 mg/L for *P. aeruginosa*), while amikacin and the carbapenems also possessed good activity against many Gram-negative pathogens. Linezolid, tigecycline and vancomycin susceptibility were stable over the course of this study, but ampicillin, piperacillin-tazobactam, ceftriaxone and levofloxacin susceptibility varied by pathogen; minocycline and cefepime susceptibility decreased among several pathogens. ESBL-positive *E. coli* increased while ESBL-positive *Klebsiella* spp., vancomycin-resistant enterococci and MRSA decreased in prevalence during this study.
